# FERPIR promotes cardiomyocyte survival and attenuates cardiac remodeling after myocardial infarction

**DOI:** 10.1038/s41419-026-08817-8

**Published:** 2026-05-21

**Authors:** Ruiquan Wang, Xinzhe Chen, Jiahao Ren, Shen Hu, Fang Liu, Luyu Zhou, Xinmin Li, Shijun Xu, Junqiang Xue, Sumin Yang, Meihua Zhang, Yunhong Wang, Kun Wang, Cuiyun Liu

**Affiliations:** 1 Key Laboratory of Maternal & Fetal Medicine of National Health Commission of China, Institute of Chronic Diseases, Shandong Provincial Maternal and Child Health Care Hospital Affiliated to Qingdao University, Qingdao University, Jinan, China; 2 Department of Neurosurgery, Beijing Hospital, National Center of Gerontology, Beijing, China; 3 Center of Diabetic Systems Medicine, Guangxi Key Laboratory of Excellence, and Department of Anatomy, Guilin Medical University, Guilin, China; 4 Department of Cardiac Surgery, Beijing Anzhen Hospital, Capital Medical University, Beijing, China; 5 Department of Rehabilitation Medicine, The Affiliated Hospital of Qingdao University, Qingdao, China; 6 Department of Cardiovascular Surgery, The Affiliated Hospital of Qingdao University, Qingdao, China; 7 Hypertension Center, Beijing Anzhen Hospital, Capital Medical University, Beijing, China

**Keywords:** Piwi RNAs, Heart failure

## Abstract

PIWI-interacting RNAs (piRNAs) are widely expressed in cardiac tissues and play important roles in cardiac pathophysiology. However, their functions and molecular mechanisms in cardiac remodeling following myocardial infarction (MI) and cardiomyocyte ferroptosis remain largely unknown. Here, we identified a ferroptosis-related piRNA (FERPIR), which inhibits ischemia/reperfusion (I/R) induced myocardial injury, cardiac remodeling and ferroptosis by targeting HNRNPA2B1-dependent regulation of Fis1. FERPIR levels were decreased in hypoxia/reoxygenation (H/R)-exposed cardiomyocytes and I/R-injured mouse hearts. FERPIR prevented I/R-induced acute injury and pathological cardiac remodeling. In vitro, overexpression of FERPIR inhibits H/R-induced ferroptosis. Mechanistically, FERPIR directly bound to HNRNPA2B1 and promoted its stability, which exhibited decreased ferroptosis and improved cardiac function upon I/R injury. Fis1 acted as a downstream regulator of HNRNPA2B1, and FERPIR recruited HNRNPA2B1 to bind to Fis1 mRNA and decreased its stability, thereby inhibiting mitochondrial fission and ferroptosis, which improves cardiac remodeling after myocardial infarction. Our findings reveal that FERPIR prevents myocardial I/R induced injury and pathological cardiac remodeling through the HNPA2B1/Fis1 axis, which provides potential therapeutic targets against cardiac injury caused by cardiomyocyte ferroptosis.

## Introduction

Myocardial ischemic/reperfusion (I/R) injury arises from the obstruction of coronary blood flow, a consequence often encountered during interventions like angioplasty for acute myocardial infarction, contrasted with treatments such as intravenous thrombolysis, heart transplantation, and coronary bypass. Cardiomyocyte death is a fundamental aspect of myocardial I/R injury, and its mechanism was previously thought to involve apoptosis [1–3], autophagy [4–6], necrosis [7, 8] and pyroptosis [9–12]. Recent studies provide compelling evidence that ferroptosis plays a key pathogenic driver in a wide range of diseases, including various cancer types, neurodegenerative diseases and inflammatory and infectious diseases [13–16]. Furthermore, studies have demonstrated ferroptosis to be a significant contributor to I/R injury, both in vivo and in vitro [17–19]. Consequently, targeting ferroptosis following I/R injury represents a crucial therapeutic approach aimed at attenuating myocardial infarction severity and minimizing subsequent cardiac damage.

PIWI-interacting RNAs (piRNAs) are small non-coding RNA molecules, typically 24 to 31 nucleotides in length, that usually bind to PIWI proteins to regulate cellular pathways. PiRNAs are expressed in a tissue-specific manner across various human tissues. Notably, abnormal piRNA expression in cardiac tissues highlights them as novel biomarkers and therapeutic targets for cardiovascular diseases [1, 20, 21]. Emerging evidence demonstrates that piRNAs are widely expressed in the cardiovascular system and actively contribute to the pathogenesis of cardiovascular disorders, including apoptosis [1, 22] and necrosis [23]. Consequently, these findings establish piRNAs as crucial regulators of programmed cell death. However, the precise role of piRNAs in regulating ferroptosis remains largely uncharacterized. While previous studies have demonstrated piRNAs’ involvement in regulating ferroptosis in the liver [24] and hepatocellular carcinoma [25], their specific role in cardiomyocyte ferroptosis and the underlying mechanisms are poorly understood.

HNRNPA2B1 exerts crucial regulatory effects in cardiovascular diseases through RNA-protein interactions. A previous study demonstrated that HNRNPA2B1 enhances PFN2 mRNA stability by recognizing and binding to its m6A site, thereby regulating PFN2/FTH1 pathway-mediated myocardial ferroptosis [22, 26]. HNRNPA2B1 participates in pulmonary vascular remodeling via protein-protein interactions, regulating vascular smooth muscle cell proliferation and macrophage polarization to modulate monocrotaline-induced pulmonary hypertension [27]. Furthermore, HNRNPA2B1 interacts with the arginine demethylase JMJD6, regulating its own dynamic modification status. These modifications critically influence HNRNPA2B1’s binding efficiency to target RNAs, such as mRNAs encoding inflammatory factors and vasoactive substances, thereby impacting downstream signaling pathways [27, 28]. Nonetheless, little is known about whether HNRNPA2B1 can bind with non-coding RNAs to jointly regulate downstream target RNA stability.

In the present study, we demonstrated that FERPIR is involved in the regulation of cardiomyocyte ferroptosis and prevents myocardial ischemia-reperfusion-induced acute injury and pathological cardiac remodeling. We found that FERPIR physically binds to HNRNPA2B1 and enhances its protein stability, consequently reducing Fis1 mRNA stability and suppressing mitochondrial fission. Our findings uncover a previously unrecognized piRNA-mediated regulatory network governing cardiac remodeling following myocardial infarction and elucidate a novel therapeutic mechanism for piRNA-based intervention in cardiovascular diseases.

## Methods

Detailed methodological protocols are provided in the Supplementary Materials. All experimental datasets generated in this study are available upon reasonable request from the corresponding authors, who will evaluate and fulfill data-sharing requests in accordance with institutional guidelines.

### Neonatal mouse cardiomyocytes isolation and culture

Primary cardiomyocytes were isolated from 1-3-day-old neonatal mice. Hearts were aseptically harvested and placed in a 6-well plate. All cardiac tissues underwent three washes with ice-cold phosphate-buffered saline (PBS) before being minced into 1 mm³ fragments using sterile surgical blades. Heart tissue digestion was performed using 5 mL of PBS containing 0.14 mg/mL collagenase II and 1.2 mg/mL pancreatin at 37 °C with continuous agitation, with the digestion process repeated until complete tissue dissociation. The resulting cell suspension was transferred to a 50 mL conical tube containing 5 mL of heat-inactivated horse serum (Gibco) for enzyme neutralization, followed by centrifugation at 1000 rpm for 5 minutes. The cell pellet was subsequently resuspended in complete culture medium consisting of DMEM/F12 (Servicebio) supplemented with 5% fetal bovine serum (FBS). The cell suspension was sequentially filtered through a 100 μm nylon mesh into a 10 cm culture dish. Following 1.5 h of differential adhesion in a humidified incubator, the non-adherent cell fraction, enriched for cardiomyocytes, was collected. Primary cardiomyocytes were maintained in DMEM/F12 complete medium supplemented with 5% fetal bovine serum, 0.1 mM bromodeoxyuridine (BrdU), and 1% penicillin–streptomycin under standard culture conditions (37 °C, 5% CO₂).

To establish the H/R model, cardiomyocytes were first incubated in glucose-free DMEM and subjected to hypoxic conditions (1% O₂/94% N₂/5% CO₂) at 37 °C for 18 h in a hypoxia chamber. Subsequently, cells were reoxygenated by replacing the medium with complete growth medium (containing 5 mM glucose) and transferring to normoxic conditions (95% air/5% CO₂) at 37 °C for 6 h.

### Animal studies

Eight- to ten-week-old male C57BL/6 mice were obtained from Jinan Pengyue Laboratory Animal Breeding Co., Ltd. (Shandong, China). All experimental procedures were conducted in accordance with the NIH Guide for the Care and Use of Laboratory Animals and were approved by the Institutional Animal Care and Use Committee of Qingdao University (approval number: c577620210617068). Mice were randomly assigned to experimental groups using a computer-generated randomization scheme, with investigators blinded to group allocation through numerical coding of animals and samples. No animals were excluded, and analyses were restricted to mice with comparable body weights and normal physiological indices during surgical procedures.

The myocardial I/R injury model of adult mice was established by ligating the left anterior descending coronary artery (LAD). The specific operation is as follows: the mice were anesthetized and intubated, and the animal ventilator was used to assist the normal breathing of the mice. The mouse thorax was opened in a suitable position, and most of the heart was exposed using a chest expander. LAD was ligated at 2.5 mm distal to the ascending aorta with a nylon suture (6-0, Ningbo Medical Suture Co., Ltd., China) to establish an adult mouse myocardial I/R injury model. During LAD surgery, ensure the integrity of the pericardial structure and properly close the thoracic incision and skin wound. After the operation, the mice were placed on a heat preservation board for recovery. In the sham group, LAD ligation was not performed, and the other surgical procedures were the same.

To investigate the role and functional relevance of FERPIR in acute myocardial ischemia-reperfusion injury and subsequent cardiac remodeling, we established a murine I/R model. FERPIR overexpression was achieved by administering agomir via tail vein injection. For acute I/R injury studies, agomir was injected (15 mg/kg) three days before inducing injury (30 min coronary ligation followed by 24 h reperfusion). To assess FERPIR’s impact on I/R-induced pathological cardiac remodeling, mice underwent a longer reperfusion period (3 weeks), with agomir administered (15 mg/kg) seven days prior to injury induction and weekly thereafter for three consecutive weeks. Control mice received an equal volume of scrambled agomir.

### Statistical analysis

All quantitative data are expressed as mean ± standard deviation (SD). Statistical analyses were performed using GraphPad Prism 9.0 (GraphPad Software). For comparisons between two groups, unpaired two-tailed Student’s *t* tests were applied. Multiple group comparisons were analyzed by one-way ANOVA followed by Tukey’s post hoc test. For factorial experimental designs, two-way ANOVA with Sidak correction was employed. All experiments included at least five independent biological replicates showing consistent results. Statistical significance was defined as *P* < 0.05.

## Results

### Identification and characterization of piRNAs associated with cardiac ferroptosis

**Fig. 1:**
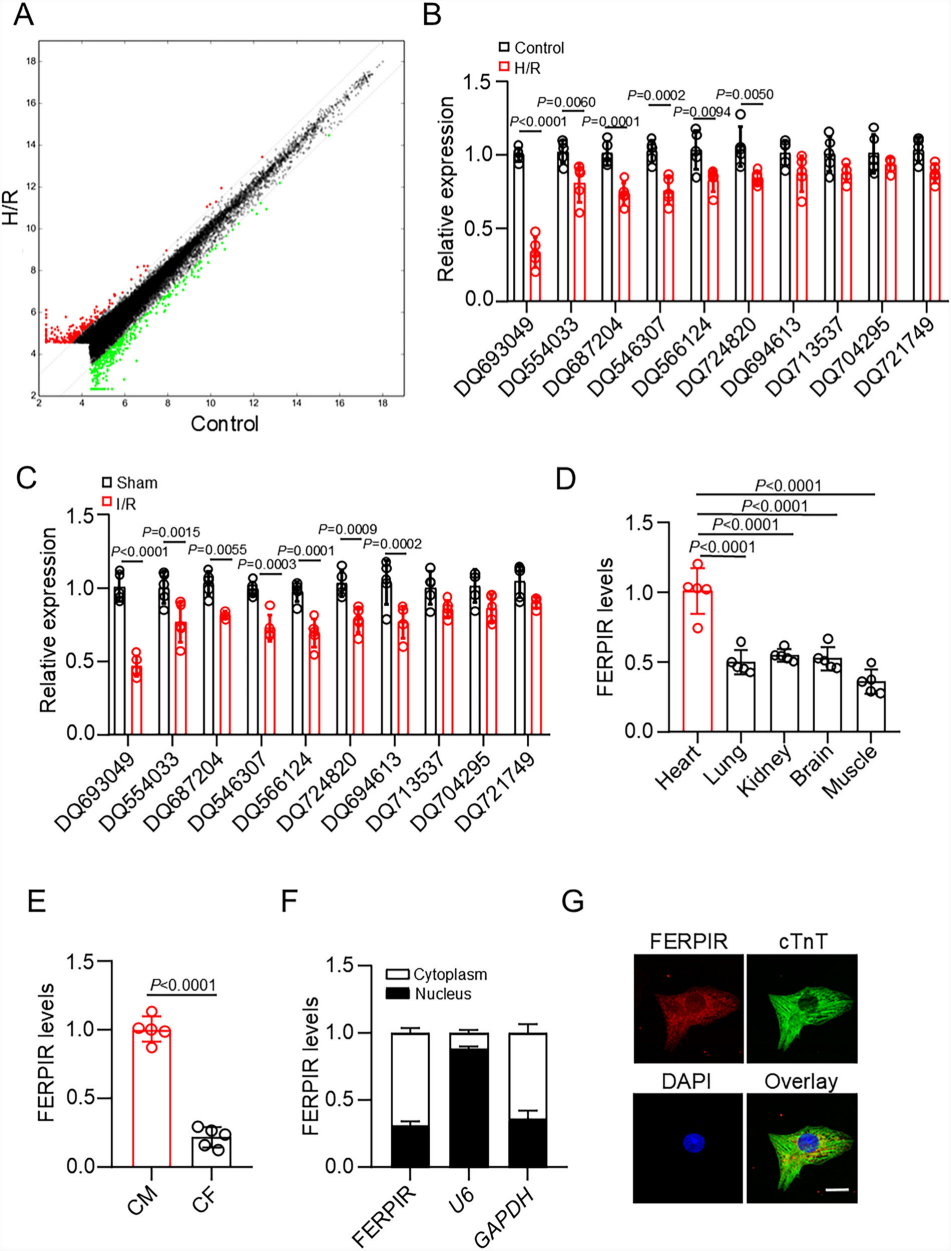
Identification and characterization of cardiac ferroptosis-associated piRNAs. **A** PiRNA microarray analysis was used to profile piRNA expression in Control and H/R-treated cardiomyocytes. Red dots represent upregulated genes, and green dots represent downregulated genes. **B** Relative expression levels of 10 downregulated candidate piRNAs were analysed by RT-qPCR in the control and H/R treated cardiomyocytes (*n* = 5 independent experiments). **C** Relative expression levels of 10 downregulated candidate piRNAs were analysed by RT-qPCR in the hearts of mice from the sham and I/R groups (*n* = 5 mice per group). **D** Relative expression levels of FERPIR were detected by RT-qPCR in mouse heart, lung, kidney, brain, and muscle tissues, with the highest expression level in the heart (*n* = 5 mice per group). **E** Relative expression levels of FERPIR were detected by RT-qPCR in cardiomyocytes and cardiac fibroblasts, with significantly higher expression in cardiomyocytes (*n* = 5 independent experiments). **F** After nuclear-cytoplasmic separation of cardiomyocytes, the distribution of FERPIR in the nucleus and cytoplasm was detected by RT-qPCR, and *U6* (nuclear reference) and *GAPDH* (cytoplasmic reference) were used to verify the purity of separation. FERPIR was expressed in both the cytoplasm and nucleus (*n* = 6 independent experiments). **G** Fluorescence in situ hybridization and immunofluorescence co-localization images showing the expression and localization of FERPIR in cardiomyocytes. FERPIR was labeled with a probe (red), cardiomyocytes were labeled with cardiac troponin T (cTnT, green), and cell nuclei were labeled with DAPI (blue). Scale bar, 20 μm. Data are presented as mean ± SD. Unpaired t-test was used for comparisons between two groups (Fig. 1E), one-way ANOVA followed by Tukey’s multiple comparison test was used for multiple group comparisons (Fig. 1D), and two-way ANOVA followed by Sidak’s multiple comparison test was used for factorial designs (Fig. 1B, C). A *P*-value < 0.05 was considered statistically significant.

To systematically identify ferroptosis-related piRNAs in cardiomyocytes, we performed piRNA microarray analysis (Supplementary Table 1) under hypoxia/reoxygenation (H/R) conditions (Fig. 1A). Among the differentially expressed piRNAs, we selected the top 20 most dysregulated (10 upregulated and 10 downregulated) and validated their expression by RT-qPCR (Fig. 1B and Supplementary Fig. 1A). We further examined the expression of these piRNAs in ischemia/reperfusion (I/R)-injured mouse hearts. Notably, DQ693049 showed significantly reduced expression in both H/R-treated cardiomyocytes and I/R-injured hearts (Fig. 1C and Supplementary Fig. 1B). Given these results implicating DQ693049 in I/R-induced cardiac injury, we selected this piRNA for further characterization and designated it as ferroptosis-associated piRNA (FERPIR). Subsequently, we analyzed FERPIR expression across multiple tissues and found it was predominantly expressed in the heart compared to other organs (Fig. 1D). We isolated the two primary cardiac cell types and found FERPIR expression was significantly higher in cardiomyocytes than fibroblasts (Fig. 1E). Subcellular localization analysis showed FERPIR expression in both nuclear and cytoplasmic compartments (Fig. 1F, G), and H/R treatment decreased FERPIR levels (Supplementary Fig. 1C). These findings indicate that I/R injury or H/R treatment induces FERPIR downregulation, suggesting its potential role in regulating cardiomyocyte death.

### FERPIR protects from acute myocardial I/R injury and ferroptosis

**Fig. 2:**
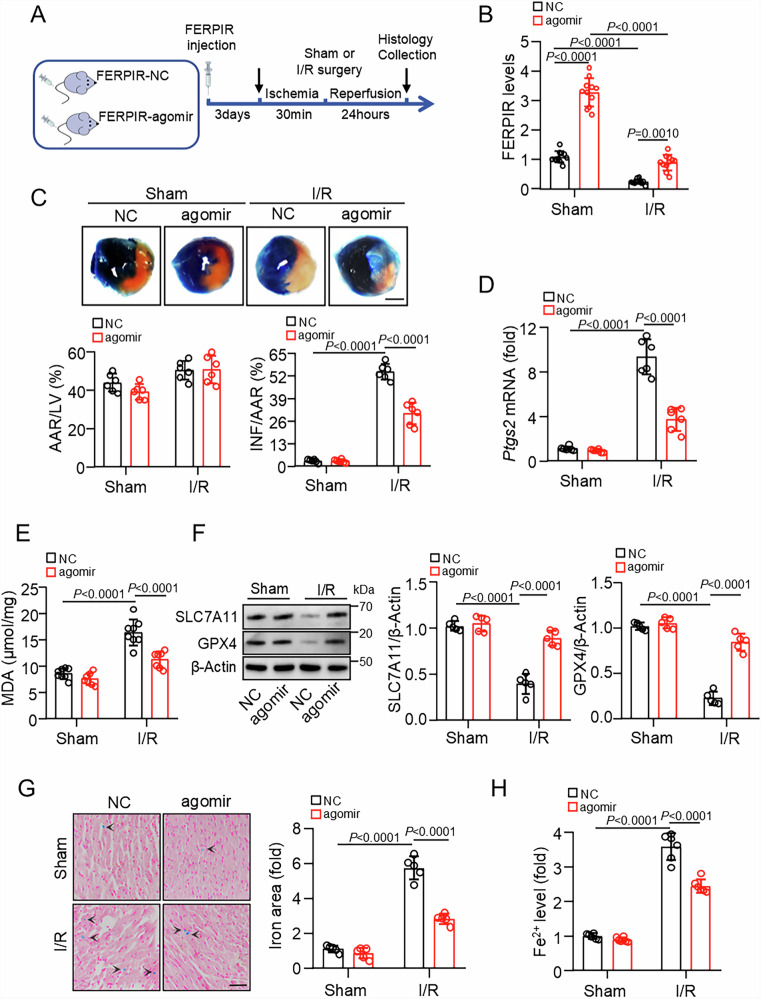
FERPIR against cardiac I/R injury and ferroptosis in vivo. **A** Mice were injected intravenously with FERPIR-agomir or a negative control (FERPIR-NC). Three days post-injection, animals underwent either sham surgery or left anterior descending (LAD) coronary artery ligation for 30 min, followed by 24 h of reperfusion. Cardiac tissues were then collected for analysis. **B** FERPIR expression in myocardial tissues. RT-qPCR was performed to detect the expression level of FERPIR in agomir or NC-injected heart (*n* = 11 mice per group). **C** Evans Blue-TTC double staining was used to quantify the area at risk/left ventricular area (AAR/LV, %), and the infarct size/area at risk (INF/AAR, %) (*n* = 6 mice per group). Scale bar, 3 mm. **D** Relative expression level of *Ptgs2* mRNA in cardiac tissues (*n* = 6 mice per group). **E** MDA content in cardiac tissues (*n* = 8 mice per group). **F** WB analysis and quantification of SLC7A11 and GPX4 protein expression, with β-Actin as an internal control (*n* = 5 mice per group). **G** Prussian blue staining images showing iron deposition in cardiac tissues, with blue spots representing iron deposition (*n* = 5 mice per group). Scale bar, 25 μm. **H** Fe²⁺ content in cardiac tissues (*n* = 6 mice per group). Data are presented as mean ± SD. Two-way ANOVA followed by Sidak’s multiple comparison test was used for statistical analysis (Fig. 2B–H). A *P*-value < 0.05 was considered statistically significant.

To investigate FERPIR’s in vivo function, we used a murine model of cardiac I/R injury. Mice were injected with agomir, which mediated FERPIR overexpression (FERPIR-agomir), and then subjected to acute myocardial I/R injury (30 min coronary ligation and 24 h reperfusion) (Fig. 2A). Mouse hearts were harvested after I/R, and the expression of FERPIR was significantly increased in mice hearts treated with agomir (Fig. 2B and Supplementary Fig. 2A), together with a remarkable decrease in the infarct size as observed by Evans blue-TTC staining (Fig. 2C). Our initial assessment revealed that FERPIR overexpression did not influence I/R-induced apoptosis (Supplementary Figs. 2B, C). However, subsequent experiments demonstrated that FERPIR overexpression significantly attenuated I/R injury-induced ferroptosis, as confirmed by changes in key ferroptosis-related molecules. FERPIR overexpression suppressed I/R-induced *Ptgs2* mRNA (Fig. 2D and Supplementary Fig. 3A) and COX-2 protein expression (Supplementary Fig. 3B). Concurrently, FERPIR reduced I/R-induced MDA and 4-HNE levels (Fig. 2E and Supplementary Fig. 3C), while increasing the mRNA and protein expression of SLC7A11 and GPX4 (Supplementary Fig. 3D, E and Fig. 2F). FERPIR overexpression attenuated I/R-induced iron accumulation, as evidenced by reduced Prussian blue staining of iron (Fig. 2G) and lower cellular Fe^2+^ content (Fig. 2H) in FERPIR-overexpressing mice. Together, these findings demonstrate that FERPIR mitigates acute I/R injury by reducing myocardial infarct size and alleviating ferroptosis.

### FERPIR prevents I/R-induced pathological cardiac remodeling

**Fig. 3:**
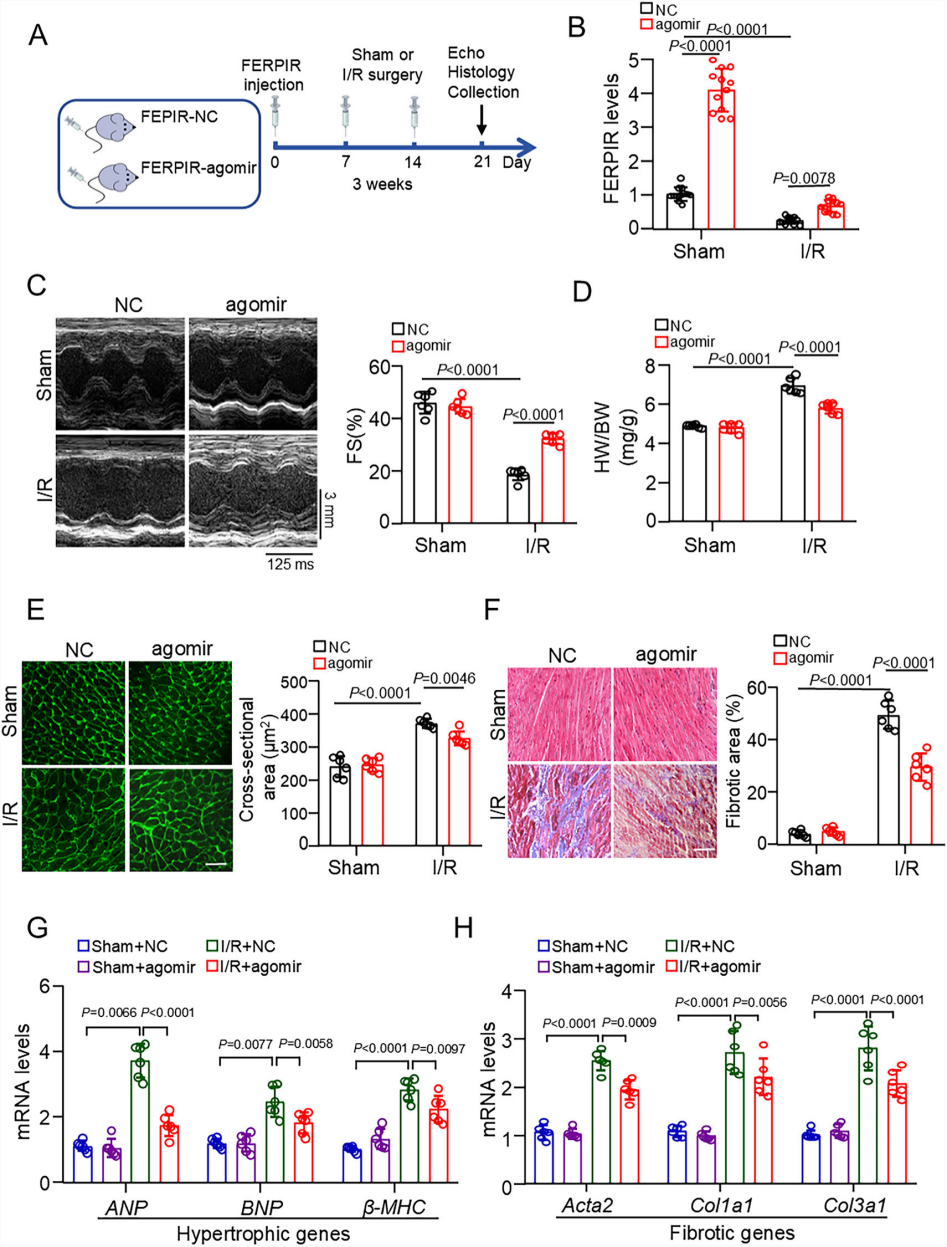
FERPIR prevents I/R-induced pathological cardiac remodeling. **A** Schematic diagram of the experimental procedure: mice were injected with FERPIR-agomir or NC via the tail vein, and 7 days later, sham surgery or I/R surgery was performed. Transthoracic echocardiography was performed, and tissues were collected 3 weeks after surgery. **B** RT-qPCR was performed to detect the expression level of FERPIR in agomir or NC-injected hearts (*n* = 12 mice per group). **C** Transthoracic echocardiography was used to detect left ventricular fractional shortening (FS, %) (*n* = 6 mice per group). Scale bar, 3 mm; Time, 125 ms. **D** Heart weight/body weight (HW/BW) ratio (*n* = 6 mice per group). **E** Wheat germ agglutinin (WGA) staining images showing cardiomyocyte cross-sectional area (*n* = 6 mice per group). Scale bar, 20 μm. **F** Masson’s trichrome staining images showing myocardial fibrosis, with blue areas representing fibrotic tissue (*n* = 6 mice per group). Scale bar, 50 μm. **G** RT-qPCR was performed to detect the expression levels of hypertrophy-related genes (*ANP, BNP, β-MHC*) (*n* = 6 mice per group). **H** RT-qPCR was performed to detect the expression levels of fibrosis-related genes (*Acta2, Col1a1, Col3a1*) (*n* = 6 mice per group). Data are presented as mean ± SD. Two-way ANOVA followed by Sidak’s multiple comparison test was used for factorial designs (Fig. 3B–H). A *P*-value < 0.05 was considered statistically significant.

We subsequently investigated the functional relevance of FERPIR in I/R-induced pathological cardiac remodeling, and agomir was delivered into the tail veins of mice to overexpress FERPIR (FERPIR-agomir). Seven days after FERPIR injection, I/R and sham surgery were conducted for 3 weeks (Fig. 3A). The efficiency of FERPIR overexpression in mouse myocardial tissue was verified by RT-qPCR (Fig. 3B). FERPIR-agomir treatment significantly improved cardiac function compared to the FERPIR-NC group, as evidenced by increased fractional shortening (FS) (Fig. 3C). Furthermore, FERPIR injection enhanced post-I/R survival rates, demonstrating its cardioprotective effects (Supplementary Fig. 3F). FERPIR overexpression in mice led to significantly reduced heart weight/body weight ratios (Fig. 3D) and cardiomyocyte cross-sectional areas (Fig. 3E) compared to NC-treated mice following I/R injury. Moreover, FERPIR overexpression attenuated I/R-induced cardiac fibrosis, as evidenced by Masson trichrome staining (Fig. 3F). This protective effect was further supported by the reduced expression of hypertrophic genes (Fig. 3G) and fibrosis-associated genes (Fig. 3H) upon I/R-induced remodeling. Collectively, FERPIR overexpression attenuates I/R-induced cardiac dysfunction and fibrosis.

### FERPIR inhibits H/R-induced ferroptosis in cardiomyocytes

**Fig. 4:**
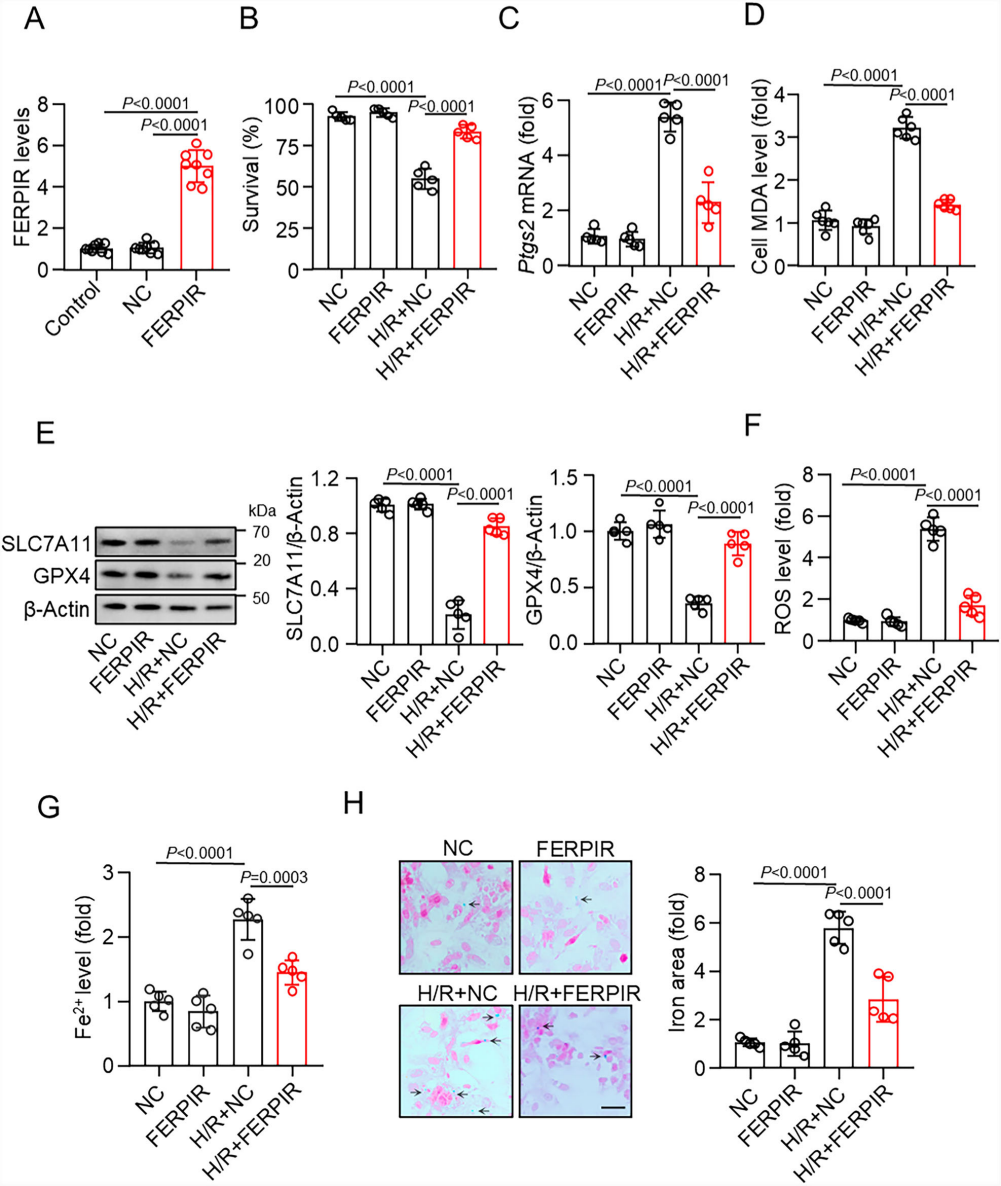
FERPIR inhibits ferroptosis and improves cardiomyocyte survival. **A** Verification of FERPIR transfection efficiency after transfecting FERPIR agomir into cardiomyocytes. The expression of FERPIR was analysed by RT-qPCR (*n* = 8 independent experiments). **B** CCK-8 assay was used to detect the survival rate of cardiomyocytes (*n* = 5 independent experiments). **C** Relative expression levels of *Ptgs2* were analysed by RT-qPCR in cardiomyocytes (*n* = 5 independent experiments). **D** Relative MDA content in cardiomyocytes (*n* = 6 independent experiments). **E** Western blot analysis and quantification of SLC7A11 and GPX4 protein expression in cardiomyocytes, with β-Actin as an internal control (*n* = 5 independent experiments). **F** Bar graph showing the relative ROS level in cardiomyocytes, detected with the C11 BODIPY 581/591 probe (*n* = 5 independent experiments). **G** Intracellular Fe²⁺ level in cardiomyocytes (*n* = 5 independent experiments). **H** Prussian blue staining images showing iron deposition in cardiomyocytes, with blue spots representing iron deposition (*n* = 5 independent experiments). Scale bar, 25 μm. Data are presented as mean ± SD. One-way ANOVA followed by Tukey’s multiple comparison test was used for statistical analysis (Fig. 4A–H). A *P*-value < 0.05 was considered statistically significant.

To verify FERPIR’s protective role against ferroptosis in cardiomyocytes, we transfected FERPIR agomir into these cells and confirmed its overexpression by RT-qPCR (Fig. 4A). FERPIR treatment effectively reduced H/R-induced cell death (Supplementary Fig. 4A) and promoted cell survival (Fig. 4B). FERPIR inhibited H/R-induced ferroptosis, evidenced by decreased *Ptgs2* expression (Fig. 4C) and MDA levels (Fig. 4D). Furthermore, FERPIR overexpression led to increased SLC7A11 and GPX4 protein expression (Fig. 4E) and reduced lipid ROS production (Fig. 4F). Consistent with these findings, cellular Fe^2+^ elevation (Fig. 4G) and iron deposition (Fig. 4H) were also suppressed by FERPIR following H/R.

We transfected cardiomyocytes with FERPIR antagomir (anta) and confirmed its knockdown efficiency by RT-qPCR (Supplementary Fig. 4B). FERPIR knockdown promoted ferroptosis, evidenced by elevated *Ptgs2* expression (Supplementary Fig. 4C) and reduced SLC7A11 and GPX4 protein expression (Supplementary Fig. 4D). Moreover, FERPIR inhibition in cardiomyocytes elevated cellular Fe²⁺ levels (Supplementary Fig. 4E) and iron deposition (Supplementary Fig. 4F), as detected by Prussian blue staining. Crucially, the ferroptosis inhibitor Ferrostatin-1 (Fer-1) rescued FERPIR knockdown-induced ferroptosis (Supplementary Fig. 5A, B), confirming the specific role of FERPIR in regulating ferroptosis. Collectively, these findings demonstrate that FERPIR acts as a negative regulator of ferroptosis and protects cardiomyocytes against H/R-induced ferroptosis.

### FERPIR interacts with HNRNPA2B1 and promotes its protein stability

**Fig. 5:**
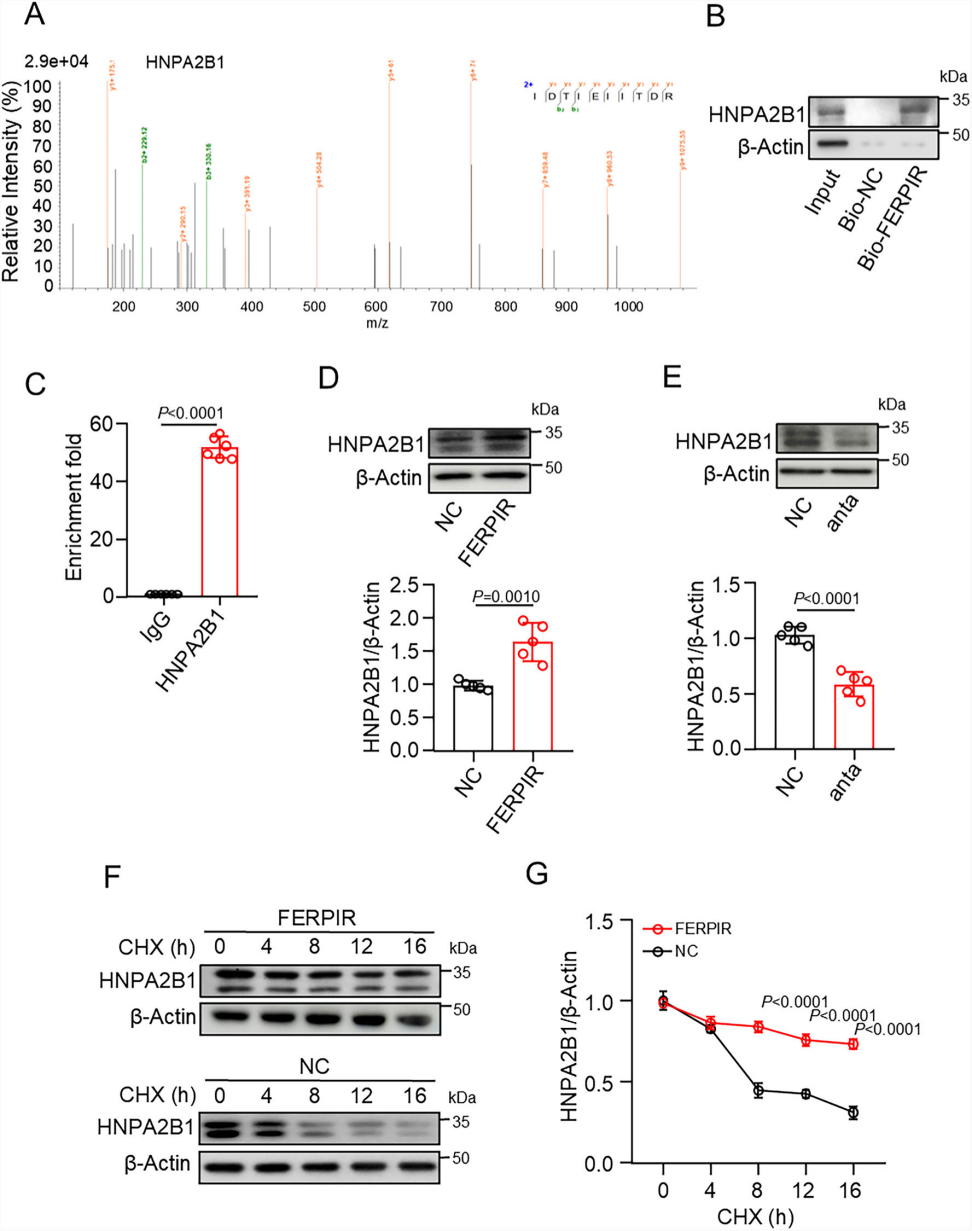
FERPIR interacts with HNRNPA2B1 and maintains its stability. **A** HNRNPA2B1 was identified by MS in the biotinylated FERPIR group. **B** RNA pull-down assay to verify the Bio-FERPIR group could specifically enrich HNPA2B1, while the Bio-NC group had no enrichment. **C** RIP assay verifying the binding between FERPIR and HNPA2B1 (*n* = 6 independent experiments). **D** FERPIR agomir (agomir) was transfected into cardiomyocytes. WB analysis and quantification of HNPA2B1 protein level showed it was significantly higher than that in the NC group (*n* = 5 independent experiments). **E** FERPIR antagomir (anta) was transfected into cardiomyocytes. WB analysis and quantification of HNPA2B1 protein level showed it was significantly lower than that in the NC group (*n* = 5 independent experiments). **F**, **G** Cycloheximide (CHX, 100 μg/mL) chase assay: HNPA2B1 protein expression was detected at 0, 4, 8, 12, and 16 h respectively. The degradation rate of HNPA2B1 was significantly slowed down, and the half-life was extended in the FERPIR overexpression group (*n* = 5 independent experiments). Data are presented as mean ± SD. An unpaired t-test was used for comparisons between two groups (Fig. 5C–E), and two-way ANOVA followed by Sidak’s multiple comparison test was used for factorial designs (Fig. 5G). A *P*-value < 0.05 was considered statistically significant.

To elucidate how FERPIR regulates myocardial injury, we conducted RNA pull-down assays using biotinylated FERPIR in cardiomyocytes, followed by LC-MS/MS to identify interacting proteins (Supplementary Table 2). Among the identified FERPIR-interacting proteins, we focused on HNRNPA2B1 (HNPA2B1) (Fig. 5A), as its role in ferroptosis regulation was previously unknown. Direct interaction between FERPIR and HNPA2B1 in cardiomyocytes was further confirmed by both RNA pull-down (Fig. 5B) and RIP assays (Fig. 5C).

Further studies revealed that FERPIR overexpression enhanced (Fig. 5D) and FERPIR knockdown decreased (Fig. 5E) HNPA2B1 protein expression in cardiomyocytes. Cycloheximide (CHX) chase assays revealed that FERPIR overexpression significantly extended HNPA2B1’s half-life (Fig. 5F, G), demonstrating FERPIR’s role in stabilizing its binding partner rather than regulating its expression. These results indicate that FERPIR interacts with HNPA2B1 and preserves the stability of the HNPA2B1 protein. To investigate the interaction between FERPIR and PIWI proteins, we employed pull-down (Supplementary Fig. 5C) and RIP assays (Supplementary Fig. 5D) to specifically demonstrate FERPIR’s association with PIWI-like protein 4 (PIWIL4) in cardiomyocytes. Consequently, we propose that the FERPIR-PIWIL4 complex interacts with HNPA2B1, thereby potentially regulating ferroptosis.

### HNPA2B1 exerts a protective effect against ferroptosis in cardiomyocytes

**Fig. 6:**
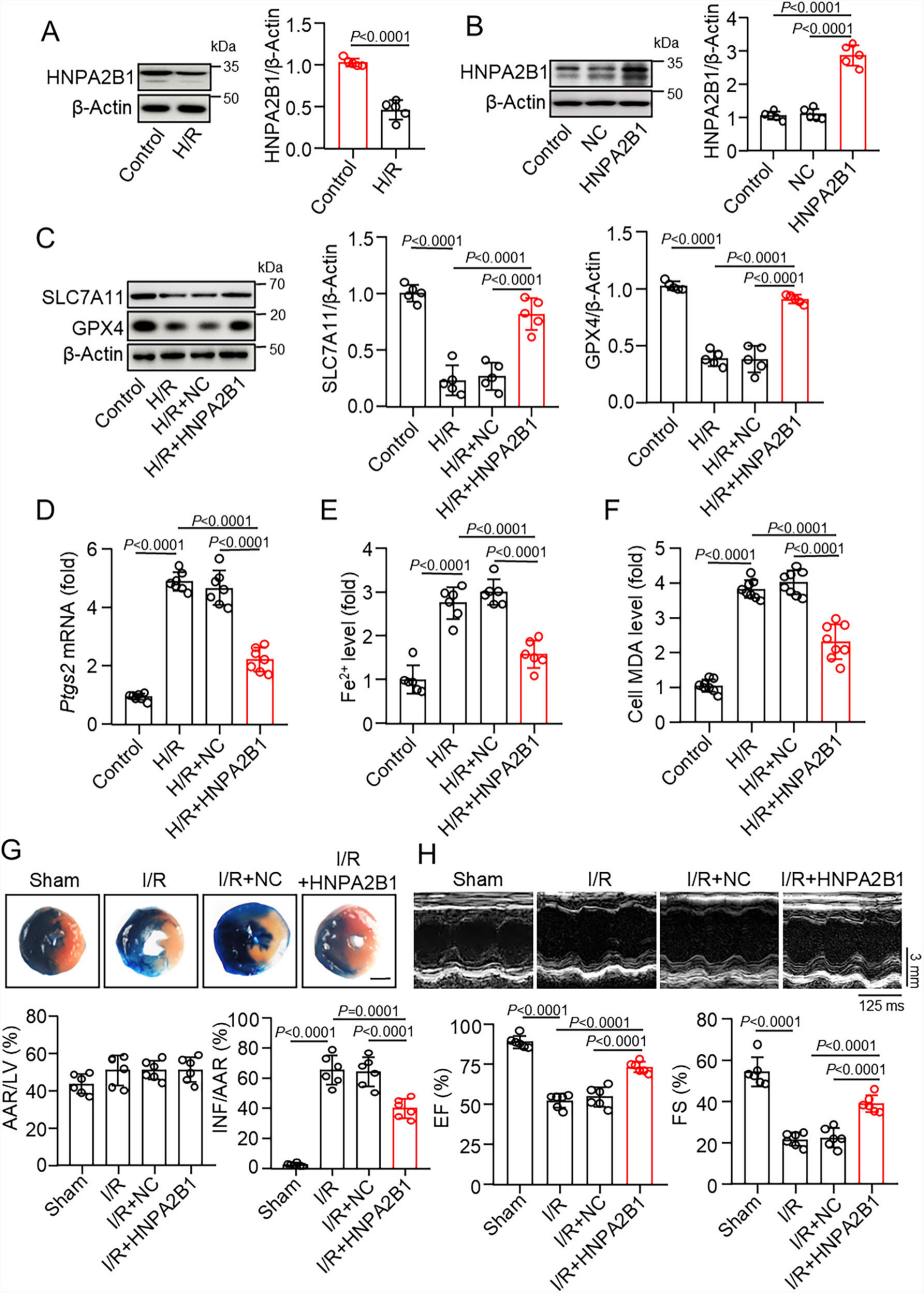
HNPA2B1 exerts a protective effect against ferroptosis in cardiomyocytes. **A** An H/R injury model was established in cardiomyocytes. WB analysis and quantification showed that the protein level of HNPA2B1 in cardiomyocytes (*n* = 5 independent experiments). **B** HNPA2B1 virus was transfected into cardiomyocytes. WB verification of HNPA2B1 overexpression efficiency showed that the protein level of HNPA2B1 in cardiomyocytes was significantly increased, with β-Actin as an internal control (*n* = 5 independent experiments). **C** WB analysis and quantification of SLC7A11 and GPX4 protein expression (*n* = 5 independent experiments). **D** Relative expression levels of *Ptgs2* mRNA were analysed by RT-qPCR in cardiomyocytes (*n* = 7 independent experiments). **E** Fe²⁺ levels in cardiomyocytes (*n* = 6 independent experiments). **F** Relative MDA content in cardiomyocytes (*n* = 8 independent experiments). **G** Evans Blue-TTC double staining was used to quantify the area at risk/left ventricular area (AAR/LV, %), and the infarct size/area at risk (INF/AAR, %) (*n* = 6 mice per group). Scale bar, 3 mm. **H** Transthoracic echocardiography was used to detect ejection fraction (EF, %) and fractional shortening (FS, %) (*n* = 6 mice per group). Scale bar, 3 mm; Time, 125 ms. Data are presented as mean ± SD. Unpaired t-test was used for comparisons between two groups (Fig. 6A), one-way ANOVA followed by Tukey’s multiple comparison test was used for multiple group comparisons (Fig. 6B–H). A *P*-value < 0.05 was considered statistically significant.

Next, we investigated whether HNPA2B1 is involved in H/R- or I/R-mediated ferroptosis, we first detected the expression of HNPA2B1 and found that the expression levels of HNPA2B1 were decreased by H/R (Fig. 6A). Overexpression of HNPA2B1 (Fig. 6B) inhibited H/R-induced ferroptosis as demonstrated by increased SLC7A11 and GPX4 protein expression (Fig. 6C), decreased *Ptgs2* expression (Fig. 6D), reduced cellular Fe²⁺ levels and MDA levels (Fig. 6E, F). In addition, knockdown of HNPA2B1 (siHNPA2B1) (Supplementary Fig. 6A) promoted ferroptosis as indicated by decreased SLC7A11 and GPX4 protein expression (Supplementary Fig. 6B, C), increased cellular Fe²⁺ levels (Supplementary Fig. 6D) and MDA levels (Supplementary Fig. 6E).

In vivo, HNPA2B1 expression was downregulated following I/R surgery (Supplementary Fig. 6F). To investigate its functional role, we overexpressed HNPA2B1 in an I/R animal model and observed a significant reduction in myocardial infarction, as demonstrated by Evans blue-TTC staining (Fig. 6G). Moreover, HNPA2B1 improved cardiac function, evidenced by increased ejection fraction and fractional shortening (Fig. 6H). Overexpression of HNPA2B1 also attenuated ROS levels (Supplementary Fig. 6G) and iron deposition (Supplementary Fig. 6H) in I/R-injured hearts. Together, these findings demonstrate that HNPA2B1 modulates cardiomyocyte ferroptosis both in vitro and in vivo.

### FERPIR facilitates HNPA2B1 binding to Fis1 mRNA and suppresses its expression

**Fig. 7:**
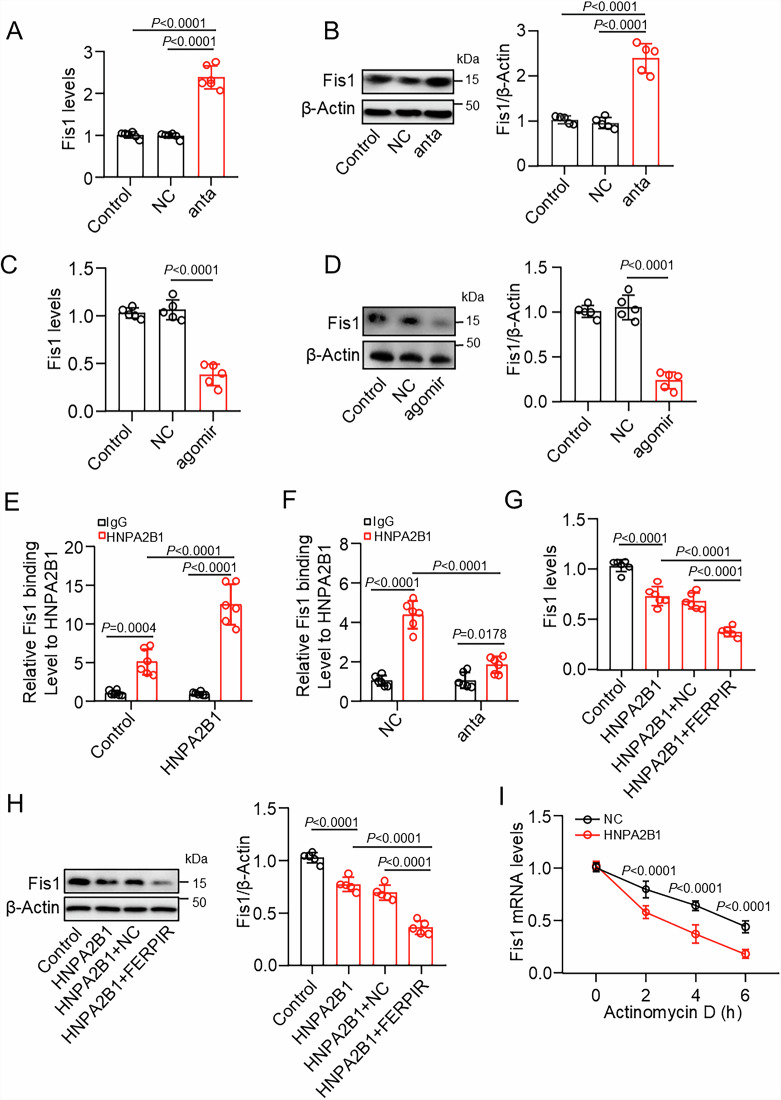
FERPIR regulates ferroptosis by the HNPA2B1/Fis1 pathway. **A** FERPIR antagomir (anta) or NC was transfected into cardiomyocytes, and the expression levels of Fis1 mRNA were analysed by RT-qPCR (*n* = 6 independent experiments). **B** WB analysis and quantification of Fis1 protein level in cardiomyocytes after FERPIR knockdown (*n* = 5 independent experiments). **C** Cardiomyocytes were transfected with FERPIR agomir, relative expression level of Fis1 mRNA was detected by RT-qPCR (*n* = 5 independent experiments). **D** WB analysis and quantification of Fis1 protein expression were performed, with β-Actin used as an internal control (*n* = 5 independent experiments). **E** RIP-qPCR analysis showing relative binding level of HNPA2B1 to Fis1 mRNA in HNPA2B1-overexpressed cardiomycytes (*n* = 6 independent experiments). **F** RIP-qPCR analysis showing relative binding level of HNPA2B1 to Fis1 mRNA in FERPIR-silenced cardiomycytes (*n* = 6 independent experiments). **G** Co-transfection of HNPA2B1 and FERPIR was performed in cardiomyocytes to achieve dual overexpression of HNPA2B1 and FERPIR. The relative expression levels of Fis1 mRNA were analysed by RT-qPCR (*n* = 6 independent experiments). **H** WB analysis and quantification of Fis1 protein level after dual overexpression of HNPA2B1 and FERPIR (*n* = 5 independent experiments). **I** Cardiomyocytes were infected with adenovirus harboring HNPA2B1 or NC, and then treated with Actinomycin D (0.1 μg/mL) for 0, 2, 4 and 6 h. The levels of Fis1 mRNA were analysed by RT-qPCR (*n* = 5 independent experiments). Data are presented as mean ± SD. One-way ANOVA followed by Tukey’s multiple comparison test was used for multiple group comparisons (Fig. 7A–D, G, H), and two-way ANOVA followed by Sidak’s multiple comparison test was used for factorial designs (Fig. 7E, F, I). A *P*-value < 0.05 was considered statistically significant.

To further identify FERPIR/HNPA2B1-mediated downstream genes, we performed a transcriptome analysis (RNA-Seq). This analysis of FERPIR-knocked down (anta) cardiomyocytes compared to control (NC) identified 1132 upregulated and 1391 downregulated differentially expressed mRNAs (one sample per group, *n* = 1) (Supplementary Fig. 7A, B). Given a previous study highlighting the crosstalk between ferroptosis and mitochondrial dynamics [29–31], we focused on Fis1 (mitochondrial fission 1 protein), a key regulator with multifaceted roles in mitochondrial dynamics. We validated Fis1 expression, observing a marked increase in Fis1 mRNA (Fig. 7A) and protein levels (Fig. 7B) following FERPIR knockdown. Conversely, FERPIR overexpression reduced Fis1 mRNA and protein expression (Fig. 7C, D) in cardiomyocytes. In parallel, I/R induced an increase in Fis1 expression (Supplementary Fig. 7C), collectively indicating FERPIR’s regulatory role in Fis1 expression.

To investigate whether HNPA2B1 is involved in FERPIR’s regulation of Fis1 mRNA expression, we performed RIP-qPCR using an anti-HNPA2B1 antibody. We found that HNPA2B1 overexpression significantly increased its binding to Fis1 mRNA (Fig. 7E). Conversely, FERPIR knockdown markedly reduced HNPA2B1 binding to Fis1 mRNA compared to controls (Fig. 7F). Furthermore, FERPIR overexpression promoted HNPA2B1-mediated effects, including reduced Fis1 mRNA levels (Fig. 7G) and decreased protein levels (Fig. 7H). We further verified that Fis1 mRNA stability was decreased in cardiomyocytes overexpressing HNPA2B1 (Fig. 7I). Collectively, these results demonstrate that FERPIR inhibits Fis1 mRNA expression by facilitating HNPA2B1 binding to Fis1 mRNA transcripts.

### FERPIR regulates mitochondrial fission and cardiomyocyte ferroptosis by targeting HNPA2B1/Fis1

**Fig. 8:**
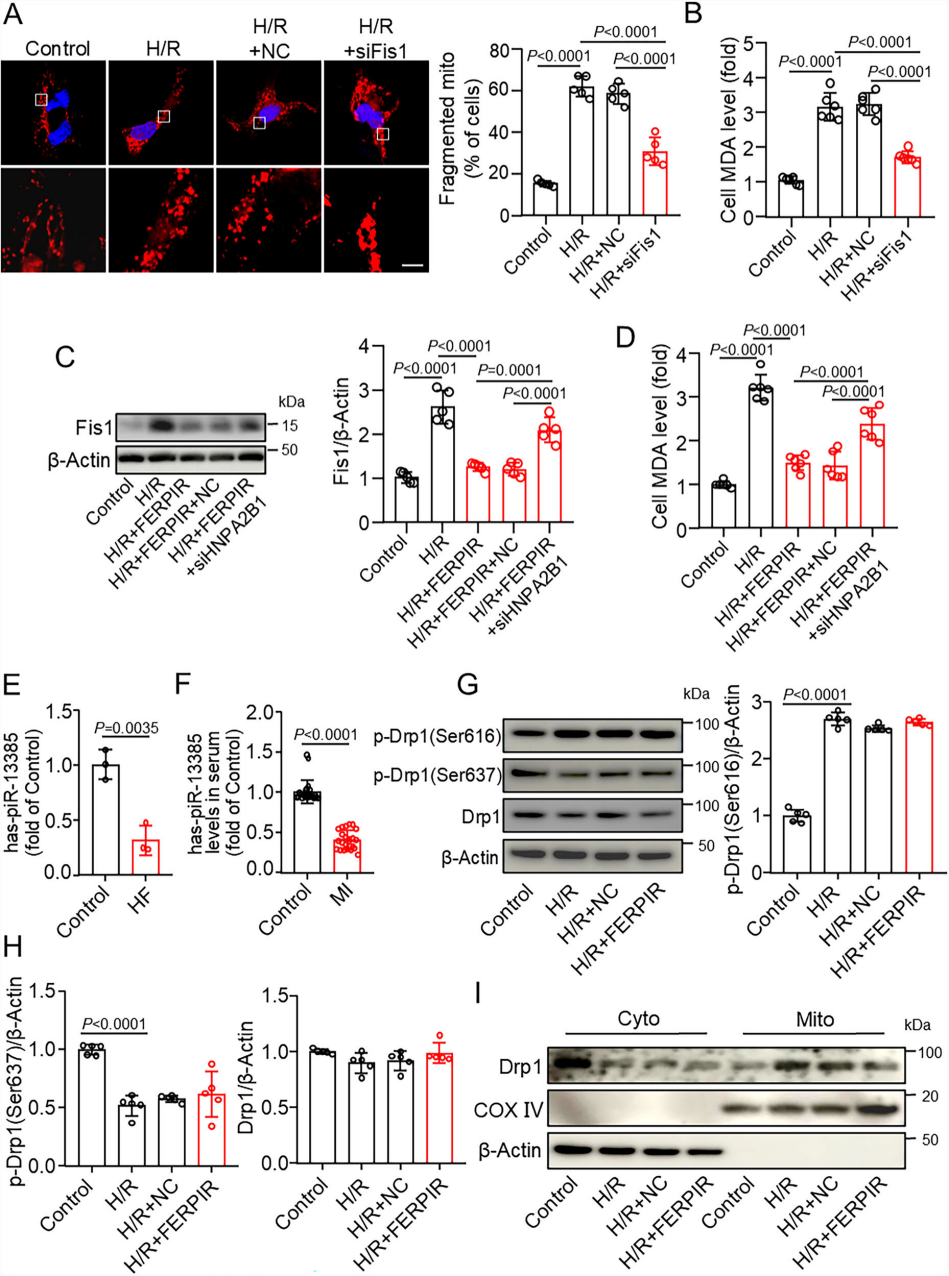
FERPIR modulates mitochondrial fission and cardiomyocyte ferroptosis by targeting HNPA2B1/Fis1. **A** Cardiomyocytes were transfected with siFis1 or NC, and then treated with H/R. Mitochondrial fission was analysed (*n* = 5 independent experiments). Mitochondria were labeled with MitoTracker (red), and cell nuclei were labeled with DAPI (blue). Scale bar, 4 μm. **B** Relative MDA content in cardiomyocytes (*n* = 6 independent experiments). **C** Cardiomyocytes were transfected with FERPIR agomir, siHNPA2B1 or NC, and then treated with H/R. WB analysis and quantification of Fis1 protein (*n* = 5 independent experiments). **D** Cardiomyocytes were treated as described in (**C**). MDA levels were analysed (*n* = 6 independent experiments). **E** The relative expression level of hsa-piR-13385 in heart failure (HF) cardiac tissues was determined by RT-qPCR (*n* = 3 patients per group). **F** The relative level of hsa-piR-13385 in serum from myocardial infarction (MI) patients was detected by RT-qPCR (*n* = 26 patients per group). **G**, **H** Cardiomyocytes were transfected with FERPIR agomir or NC, and then treated with H/R. WB analysis and quantification of p-Drp1 (Ser616), p-Drp1 (Ser637), and Drp1 protein expression were performed, with β-Actin used as an internal control (*n* = 5 independent experiments). **I** Cardiomyocytes were treated as described in (**G**). Cardiomyocytes were collected for subcellular fractionation, and WB was used to detect changes in Drp1 expression in the cytoplasm (Cyto) and mitochondria (Mito). COX Ⅳ was used as a mitochondrial marker, and β-Actin was used as a cytoplasmic marker. Data are presented as mean ± SD, and statistical differences were analyzed using one-way ANOVA followed by Tukey’s multiple comparison test (Fig. 8A–D, G, H) or an unpaired t-test was used for comparisons between two groups (Fig. 8E, F). A *P*-value < 0.05 was considered statistically significant.

We investigated Fis1’s role in mitochondrial fission and ferroptosis in cardiomyocytes. Fis1 knockdown (siFis1) attenuated H/R-induced mitochondrial fission (Fig. 8A) and significantly reduced cardiomyocyte ferroptosis. This protective effect was supported by elevated levels of anti-ferroptosis proteins (Supplementary Fig. 7D, E), decreased MDA levels (Fig. 8B), and diminished cellular Fe²⁺ (Supplementary Fig. 7F). Furthermore, Fis1 overexpression promoted mitochondrial fission (Supplementary Fig. 8A) and ferroptosis (Supplementary Fig. 8B) in cardiomyocytes. We then investigated whether FERPIR modulates mitochondrial fission and cardiomyocyte ferroptosis by targeting HNPA2B1 and Fis1. Our results demonstrated that FERPIR overexpression inhibited H/R-induced Fis1 expression (Fig. 8C) and cardiomyocyte ferroptosis (Fig. 8D). Crucially, HNPA2B1 silencing significantly reversed these effects, suggesting FERPIR acts upstream of HNPA2B1 to regulate Fis1 and ferroptosis.

We also explored the effects of FERPIR on human AC16 cells. FERPIR overexpression attenuated H/R-induced ferroptosis (Supplementary Fig. 9A–C) and mitochondrial fission (Supplementary Fig. 9D); however, these protective effects were inhibited by HNPA2B1 knockdown. Furthermore, we identified a potential human homolog of FERPIR, hsa-piR-13385. Examination of cardiac tissues revealed significantly reduced expression of hsa-piR-13385 in heart failure (HF) patients compared to healthy controls (Fig. 8E). Correspondingly, serum levels of hsa-piR-13385 were also significantly decreased in myocardial infarction (MI) patients compared to healthy subjects (Fig. 8F), highlighting its clinical relevance in cardiovascular diseases.

Fis1 functions as an adaptor protein, recruiting cytosolic dynamin-related protein 1 (Drp1) to the mitochondrial surface and promoting its oligomerization and GTPase activity, which are essential for mitochondrial constriction and division [32, 33]. To investigate Drp1’s involvement in FERPIR-mediated mitochondrial fission and cardiomyocyte ferroptosis, we assessed Drp1 translocation to mitochondria and p-Drp1 (Ser616/Ser637) levels. Our findings indicated that FERPIR did not affect Drp1 translocation or p-Drp1 levels (Fig. 8G–I, Full uncropped gel), suggesting that Drp1 is not involved in FERPIR-mediated ferroptosis. Taken together, these results indicate that Fis1 acts as a crucial downstream effector of FERPIR in modulating cardiomyocyte ferroptosis. Specifically, FERPIR exerts its cardioprotective effects by downregulating Fis1 expression. This downregulation, in turn, attenuates excessive mitochondrial fission and subsequent cardiomyocyte ferroptosis during myocardial injury.

## Discussion

PIWI-interacting RNAs (piRNAs), a class of small non-coding RNAs originally identified for their crucial roles in germline development and transposon silencing, are now increasingly recognized for their functional significance in somatic tissues, including those relevant to cardiovascular health and disease [1, 20, 23, 34]. Our previous study has demonstrated that piRNAs manipulate cardiomyocyte apoptosis [1] and necrosis [23]. Furthermore, other studies have confirmed that piRNAs play a role in regulating cardiomyocyte apoptosis [22]. Collectively, these findings establish piRNAs as crucial regulators of I/R-induced programmed cell death. Previous studies have established ferroptosis as a significant contributor to I/R injury, both in vivo and in vitro [17–19]. However, the precise role of piRNAs in regulating ferroptosis remains largely unexplored. Hsa_piR_016975 promotes hepatocellular carcinoma (HCC) growth and metastasis by targeting Maspin/GPX4 to attenuate ferroptosis [25]. Another study showed that piR-16404 attenuates environmental chemical-induced ferroptosis and liver injury by targeting CASTOR1 [24]. These studies demonstrated the role of piRNAs in regulating liver ferroptosis. However, a previous study reported that piR-000699 binds to SLC39A14 and regulates ferroptosis in aging myocardial I/R injury [35]. This research investigated the role of piRNAs in regulating ferroptosis in aging rat cardiomyocytes. Our current study builds upon this by demonstrating that FERPIR inhibits ferroptosis in non-aging mouse cardiomyocytes. Although differing in piRNA, mechanism, and species, both studies confirm the crucial role of piRNAs in ferroptosis. Future investigations will explore the effects of piRNAs on ferroptosis across various pathological models.

PiRNAs are distinguished by their association with PIWI proteins, a specialized subclass of argonaute proteins. Their canonical role involves maintaining genomic stability by silencing transposable elements (TE) via transcriptional and post-transcriptional mechanisms, such as DNA methylation and mRNA degradation [21, 36]. Beyond germline-specific TE repression, emerging evidence highlights their regulatory roles in somatic cells, including modulating gene expression, epigenetic programming, and post-transcriptional mRNA regulation [37, 38]. In our study, we identified PIWI proteins interacting with FERPIR. Subsequent RNA pull-down and RIP experiments confirmed that FERPIR binds specifically to PIWI L4, but not to PIWI L1 or PIWI L2. This suggests that FERPIR, PIWI L4, and HNPA2B1 may form a complex. This complex, in turn, could jointly regulate the stability and expression of downstream Fis1, ultimately controlling mitochondrial fission and ferroptosis. A current limitation of our research is that we have not yet determined whether the binding between PIWI L4 and HNPA2B1 is direct or indirect. Future studies will aim to elucidate their binding patterns and explore the functional consequences of their interactions.

Restricted blood flow to the myocardium triggers acute myocardial infarction (MI), the leading cause of mortality worldwide, resulting in cardiac damage and the death of viable cardiomyocytes. The gold standard treatment for MI patients is timely restoration of coronary flow, which limits infarct size. However, this intervention, known as reperfusion, paradoxically initiates a complex pathological process that also contributes to cardiac injury. Despite its sterile nature, I/R injury triggers inflammation, a process that contributes to infarct expansion and subsequent cardiac remodeling [39–42]. In the early stage of myocardial ischemia-reperfusion (I/R) injury, the surviving cardiomyocytes often undergo compensatory hypertrophy as an adaptive response to maintain cardiac contractile function. If the injury stimulus persists, compensatory hypertrophy will gradually transform into pathological hypertrophy, which is harmful and may result in the occurrence of heart failure [43, 44]. Although piRNAs have been demonstrated to participate in the regulation of I/R-induced different cardiomyocyte death types [1, 22, 23], their role in cardiac remodeling following myocardial infarction remains to be elucidated. In our current study, piRNA FERPIR protects against myocardial I/R-induced injury and pathological cardiac remodeling by regulating Fis1 in a HNPA2B1-dependent manner. Our study found that piRNA FERPIR improves cardiac remodeling after myocardial infarction by inhibiting cardiomyocyte ferroptosis, suggesting it as a potential target for the prevention and treatment of post-MI cardiac remodeling and heart failure. However, a limitation of our study is the chosen time point for the I/R-induced cardiac remodeling model. In our study, we evaluated the effect of FERPIR on cardiac remodeling 3 weeks post-I/R. Treatment with FERPIR resulted in improved I/R-induced cardiac dysfunction and reduced cardiac fibrosis. Given that cardiac remodeling and heart failure typically develop beyond 4-8 weeks post-MI [45–48], future studies will investigate the effects of FERPIR and its downstream targets on cardiac remodeling at these later time points following I/R injury.

HNPA2B1 is a widely expressed heterogeneous nuclear ribonucleoprotein (hnRNP) that functions as an RNA-binding protein, dynamically shuttling between the nucleus and cytoplasm to regulate RNA translation and stability [49, 50]. HNPA2B1 exerts multifaceted roles in RNA regulation [34] and tumor therapy [51]. In addition, in cervical cancer cells, HNPA2B1 inhibits ferroptosis by modulating FOXM1 and LCN2 expression [52]. Consistent with previous reports, our study also demonstrates HNPA2B1’s effect on ferroptosis. However, key differences emerge: the current work utilized different tissues and cell types, leading to variations in identified signaling pathways and targets. Our study reveals that HNPA2B1 inhibits I/R injury-induced cardiomyocyte ferroptosis by targeting Fis1. Specifically, HNPA2B1 binds to and destabilizes Fis1 mRNA, thereby inhibiting mitochondrial fission and subsequently ferroptosis. Further investigation is needed to determine if FOXM1 and LCN2 also act as downstream targets of HNPA2B1 in regulating I/R-induced ferroptosis in cardiomyocytes.

Fis1 is an integral outer mitochondrial membrane protein crucial for regulating mitochondrial dynamics, primarily by mediating mitochondrial fission. Dysregulation of Fis1-mediated mitochondrial fission has been implicated in various pathological conditions, including neurodegenerative diseases [53, 54], cardiovascular diseases [53, 55, 56], and certain cancers [57, 58]. Fis1 primarily functions as an adaptor protein, recruiting cytosolic dynamin-related protein 1 (Drp1) to the mitochondrial surface [32, 33]. Acting as a receptor, Fis1 facilitates Drp1’s translocation to the mitochondria, promoting its oligomerization and subsequent GTPase activity essential for mitochondrial constriction and division [59, 60]. Our study demonstrated that FERPIR inhibits cardiomyocyte ferroptosis and mitochondrial fission by targeting Fis1 in a HNPA2B1-dependent manner. To determine if Drp1 translocation and phosphorylation were involved in FERPIR-mediated mitochondrial fission and cardiomyocyte ferroptosis, we assessed Drp1 recruitment to mitochondria and p-Drp1 (Ser616/Ser637) levels. Our results showed FERPIR had no effect on Drp1 translocation or p-Drp1 levels, indicating Drp1 is not involved in FERPIR-mediated cardiomyocyte ferroptosis. Our future investigations will explore additional factors influencing mitochondrial fission, specifically examining the participation of mitochondrial fission and fusion proteins in FERPIR-regulated pathways.

## Supplementary information

Supplementary material methods

Supplementary figure

Supplementary material legends

Full uncropped gel

Supplementary Table 1

Supplementary Table 2

Supplementary Table 3
