# Supplementary material for: FERPIR promotes cardiomyocyte survival and attenuates cardiac remodeling after myocardial infarction
Source: Cell Death Dis. 2026 May 21;17(1):639. doi: 10.1038/s41419-026-08817-8 (PMC13365201; doi:10.1038/s41419-026-08817-8)
Supplement: Supplementary file 1 — Supplementary material methods [file 41419_2026_8817_MOESM1_ESM.pdf]

SUPPLEMENTARY MATERIAL METHODS

Isolation of primary cardiac fibroblasts

In the procedure for isolation and culture of mouse primary cardiomyocytes, the adherent cells were retained following 1.5 hours of differential adhesion. The supernatant was discarded, and the adherent cells were washed twice with pre-chilled phosphate-buffered saline (PBS). Subsequently, DMEM medium supplemented with 10% FBS was added, and the cells were cultured in a 37°C incubator with 5% CO_2. When the cell confluence reached 70%–80%, the cells were digested with 0.25% trypsin (containing EDTA) for 2–3 min, centrifuged at 1000 rpm for 5 min, and the resulting pellet was resuspended in complete medium. The cells were subcultured at a ratio of 1:3, and passages 2–3 (P2–P3) cells were harvested and used for subsequent experiments.

Isolation of adult mouse cardiomyocytes

Adult cardiac cardiomyocytes were isolated using the Langendorff perfusion system. The hearts of mice were removed by thoracotomy, and the aorta was isolated and connected to the Langendorff perfusion system. Hearts were perfused with Trypan's buffer (137 mM Nacl, 5.4 mM KCl, 1.2 mM MgCl2, 20 mM NaH2PO4, 20 mM HEPES, 10 mM D-Glucose, 10 mM Taurine) until the residual blood was completely pumped. Hearts were digested with collagenase type II (0.1 mg/mL, Worthington) for 15 min at 37°C until they were pale and flaccid. At the end of digestion, the digested hearts was transferred to a 6 cm dish, and the heart tissue was torn with forceps to form 3-5 mm tissue blocks. The cells were gently blown to accelerate separation, and filtered through a 100 μm filter. The collected cells were centrifuged with 200 rpm for 1 min to collect cardiomyocytes, and the supernatant was removed. The cells were resuspended using 10 mL of medium and added to culture plates containing laminin coated (5 μg/mL) for 2 h at 37°C.

Human Heart Samples

Myocardial tissue samples from human controls and heart failure patients were collected at Soochow University (Suzhou, China). This study conformed to the principles of Declaration of Helsinki and was approved by the Ethics Review Board of Soochow University (approval number: SUDA20251201H01). The control hearts were collected from victims who had been in traffic accidents without history of cardiovascular diseases from autopsy cases in the Department of Forensic Medicine, School of Basic Medical Sciences, Soochow University. The deceased's kin completed written informed consent documentation according to the Helsinki Declaration. Heart failure samples were collected and informed consent was obtained from all patients or their immediate family members prior to their participation in this study.

Human plasma samples

The ethics committees of the Beijing Anzhen Hospital affiliated to Capital Medical University approved the use of human plasma samples (approval number: KS2025016). We collected serum samples from individuals with myocardial infarction admitted to the Anzhen Hospital affiliated to Capital Medical University. We also obtained serum samples from healthy individuals with normal physical examination indicators recruited from the Anzhen Hospital affiliated to Capital Medical University. Written consent from all individuals was obtained. Plasma samples were stored at −80°C.

piRNA microarray analysis

Total RNA was extracted from the cardiomyocytes in the Control and H/R groups, and piRNA microarray analysis was performed by Kangcheng Bio-tech Inc., (Shanghai, China). The Agilent Array platform was utilized for microarray analysis. In accordance with the manufacturer's standard protocols, the labeled samples were hybridized to the Arraystar Mouse piRNA Array using Agilent's SureHyb Hybridization Chambers.

RNA sequencing

RNA-Seq high throughput sequencing and subsequent bioinformatics analysis were done by Kangcheng Bio-tech Inc., (Shanghai, China). The total RNA was extracted from antagomir or NC-transfected cardiomyocytes. The quality of the constructed libraries was assessed using an Agilent 2100 Bioanalyzer, and library quantification was performed via qPCR. The pooled libraries from different samples were sequenced on an Illumina NovaSeq 6000 sequencer. Image processing and base calling were conducted using the Solexa pipeline version 1.8.

Fluorescent in situ hybridization (FISH)

FISH assay was performed using GenePharma FISH Kit. Fixed cells were sequentially incubated with FERPIR-specific fluorescent probes at 37°C overnight, followed by washing to eliminate non-specific binding. For immunofluorescence staining of cardiomyocyte marker anti-cardiac troponin T (cTNT), cells were incubated with primary anti-cTnT antibody and corresponding fluorescent secondary antibody. After counterstaining nuclei with DAPI, images were captured via confocal microscopy to visualize FERPIR, cTnT, and nuclear localization.

Cell transfection

For neonatal mouse primary cardiomyocytes and adult mouse cardiomyocytes: First, add 125 μL of serum-free DMEM/F12 medium to a sterile EP tube. Subsequently, add 100 pmol of target nucleic acid reagents, including negative control oligonucleotide (NC), FERPIR antagomirs, FERPIR agomirs, si-Fis1, and mouse si-HNRNPA2B1, to the medium. Finally, add 4 μL of Lipo8000™ transfection reagent (Beyotime, China) and gently pipette up and down 5–8 times to mix thoroughly. Incubate the mixture at room temperature for 20 minutes to form a stable transfection complex, then evenly distribute the complex into the wells containing cardiomyocyte culture medium to ensure full coverage of the cells. Six hours

post-transfection, observe cell morphology under an inverted microscope to exclude cytotoxicity, and replace the medium with fresh complete DMEM/F12 medium supplemented with 5% fetal bovine serum and 1% penicillin-streptomycin to remove residual transfection reagents. Continue culturing for 24 hours to complete transfection, and verify overexpression or knockdown efficiency by quantitative real-time polymerase chain reaction or Western blot. The sequences and catalog numbers of the above nucleic acid reagents are as follows: FERPIR antagomirs (5'-GAAAAAAUUCUGACUUAUUGAAUGCAGAUA-3'); FERPIR agomirs (Sense: 5'-UAUCUGCAUUCAAUAAGUCAGAAUUUUUUC-3', Antisense: 5'-AAAAAUUCUGACUUAUUGAAUGCAGAUAUU-3'); si-Fis1 (Sense: 5'-GAGCACGCAAUUUGAAUAUTT-3', Antisense: 5'-AUAUUCAAAUUGCGUGCUCTT-3'); mouse si-HNRNPA2B1 (Sense: 5'-GACAAAAUUCUGCCUUAUUGAAUGCAGCUA-3', Antisense: 5'-UAGCUGCAUUCAAUAAGGCAGAAUUUUGUC-3').

Human AC16 cells were obtained from Procell (China) and transfected with FERPIR agomirs/antagomirs, human si-HNRNPA2B1 or NC. The transfection protocol for AC16 cells was consistent with that used for mouse primary cardiomyocytes. The sequence and catalog number of human si-HNRNPA2B1 are as follows: Sense: 5'-CAGAAAUACCAUACCAUCAAU-3', Antisense: 5'-AUUGAUGGUAUGGUAUUUCUG-3'. The sequences and catalog numbers of FERPIR agomirs/antagomirs and NC are the same as those used for mouse primary cardiomyocytes.

Reverse Transcription-quantitative Polymerase Chain Reaction (RT-qPCR)

Total RNA was isolated from murine cardiomyocytes or tissue samples utilizing TRIZOL reagent. The concentration and purity of the extracted total RNA were assessed with a Nanodrop One spectrophotometer. Complementary DNA (cDNA) products of the target RNA were obtained using the Evo M-MLV Reverse Transcription Kit II, with the FERPIR RT primer

(5'-GTCGTATCCAGTGCAGGGTCCGAGGTATTCGCACTGGATACGACGACA AA-3') used for reverse transcription of the FERPIR target. According to the instructions for SYBR Green RT-qPCR Mix, a 6-channel RT-qPCR instrument was used to perform two-step quantitative real-time polymerase chain reaction.

The RT-qPCR reactions were set up with the following primer sets: U6 (Forward: 5'-GCTTCGGCAGCACATATACTAA-3'; Reverse: 5'-CGCTTCACGA ATTTGCGTGTCAT-3'), GAPDH (Forward: 5'-AGGAGAGTGTTTCCTCGTCC; Reverse: 5'-TGCCGTGAGTGGAGTCATAC-3'), FERPIR (Forward: 5'-GCTGC ATTCAATAAGGCAGAAT-3'; Reverse: 5'-AGTGCAGGGTCCGAGGTATT-3'), and Ptgs2 (Forward: 5'-TGGGGGAAGAAATGTGCCAA-3'; Reverse: 5'-CAGCC ATTTCCTTCTCTCCTGT-3'). The procedure included predenaturation (95°C, 10 min), denaturation (95°C, 10 sec), annealing and extension (60°C, 30 sec; 40 cycles), and melting curve analysis (using the instrument's default collection pr ogram) to achieve amplification and detection of the target mRNA.

Echocardiography

After the operation of the mouse cardiac I/R injury model, the chest and abdomen fur of the mice was removed after mild anesthesia, and the mice were fixed on the insulation board in an upward position. Transthoracic echocardiography was performed using a VEVO 2100 imaging system equipped with a 40-MHzMS-250 scan head. Two dimensional guided M-mode images were recorded in parasternal long axis and short axis views at the level of mouse papillary muscles. The fractional shortening and ejection fraction of mouse left ventricular echocardiography were calculated with the established standard formula. All measurements consisted of more than three cardiac cycles and were averaged.

Western blot

Proteins were extracted using RIPA buffer with protease inhibitors. Lysates were centrifuged, supernatants collected. Protein concentration was determined by BCA

assay. Equal amounts were loaded onto SDS-PAGE gels, separated, then transferred to PVDF membranes. Membranes were blocked, incubated with primary/secondary antibodies, and visualized by ECL. Antibodies were used as follows: HNRNPA2B1 (Proteintech, Cat: 14813-1-AP, 1:2000); Fis1 (ABclonal, Cat: A19666, 1:2000); SLC7A11 (Zenbio, Cat: R26116, 1:2000); GPX4 (Zenbio, Cat: 381958, 1:2000); β-Actin (ABclona, Cat: AC038, 1:10,000); COX II (UpingBio, Cat: YP-mAb-02543, 1:1000); COX IV (ABclonal, Cat: A6564, 1:2000); 4-HNE (UpingBio, Cat: YP-mAb-18337, 1:500); DRP1 (UpingBio, Cat: YP-Ab-00692, 1:1000); DRP1(phospho-Ser616) (UpingBio, Cat: YP-Ab-00647, 1:1000); DRP1 (phospho-Ser637) (UpingBio, Cat: YP-Ab-00644, 1:1000); PIWIL1 (UpingBio, Cat: YP-mAb-19121, 1:1000); PIWIL2 (UpingBio, Cat: YP-mAb-08177, 1:1000); PIWIL4 (UpingBio, Cat: YP-mAb-12315, 1:1000).

Evans blue and Triphenyltetrazolium chloride (TTC) double staining

Frozen hearts were sectioned longitudinally along the left ventricle into 2-3 mm slices using a pre-cooled blade. Slices were incubated in 2% TTC at 37°C for 15-30 min, rinsed with PBS, then fixed in 4% formaldehyde. Staining yields three zones: blue (normal), red (ischemic), gray (necrotic). Infarct size was assessed by proportional area analysis of comparable sections after imaging.

Histopathological staining

Excised hearts were fixed in 4% paraformaldehyde at 4°C for 24–48 h to preserve tissue morphology and antigenicity, followed by dehydration in a graded ethanol series, clearing in xylene twice to enhance paraffin penetration, and embedding in melted paraffin at 60°C to form paraffin blocks. Five-micrometer sections were mounted on slides to prevent detachment during staining, baked at 60°C for 30 min to melt residual paraffin, deparaffinized in xylene each to remove paraffin, and rehydrated through a reverse graded ethanol series to distilled water to prepare for staining. Masson trichroic reagent (G1340; Solarbio, China) dyeing was performed

according to the manufacturer's protocol, with hematoxylin staining for nuclei, ponceau-fuchsin staining for cytoplasm and muscle fibers, and aniline blue staining for collagen fibers, enabling clear distinction between myocardial cells and fibrotic tissue. After staining, sections were dehydrated again, cleared, and mounted with neutral balsam to ensure long-term preservation of staining results for subsequent microscopic observation and image analysis.

Prussian blue staining for iron deposition detection

For cell samples, clean glass slides were placed in empty culture dishes, and cardiomyocyte suspensions were seeded onto the slides and cultured until adherent to ensure uniform cell distribution for staining. For tissue samples, mouse heart paraffin sections (5 μm thick) were prepared using a microtome, fixed with 4% paraformaldehyde for 10 minutes at room temperature to stabilize tissue structure, then dewaxed in xylene and rehydrated through graded ethanol to distilled water following standard histological procedures. Subsequent steps followed the instructions of the Prussian Blue Staining Kit (PERLS STAIN, nuclear fast red method): sections were incubated in Perl's solution (equal volumes of 2% potassium ferrocyanide and 2% hydrochloric acid) at 37°C for 30 min to form insoluble blue precipitates with ferric ions, rinsed thoroughly with distilled water to terminate the reaction, and counterstained with nuclear fast red for 5 minutes to visualize cell nuclei. Iron-positive cells (blue dots) were observed under microscope bright field, and images were captured using a digital microscope camera; ImageJ software was used to quantify the area and density of blue staining to evaluate the degree of iron deposition in cardiomyocytes or myocardial tissues.

Ferrous ions (Fe^{2+}) and Malondialdehyde (MDA) detection

Myocardial cells and tissue samples were collected, washed with phosphate-buffered saline (PBS), and lysed in cell/tissue lysis buffer. Tissues were homogenized using a tissue homogenizer to obtain a uniform homogenate. Subsequently, the samples were

centrifuged at 12,000 rpm for 15 minutes at 4°C to separate the supernatant, thereby eliminating cellular debris contamination that could interfere with the accuracy of subsequent detection. Samples were prepared using the Fe^{2+} detection kit (Elabscience) and MDA detection kit (Beyotime), respectively: for Fe^{2+} detection, supernatants were mixed with ferrous ion detection reagent in a 1:1 ratio and incubated at 37°C for 30 minutes to ensure sufficient reaction between the reagent and ferrous ions, while for MDA detection, supernatants were reacted with thiobarbituric acid (TBA) reagent in a water bath at 95°C for 40 minutes to form stable MDA-TBA adducts that enable specific quantification. After centrifugation at room temperature for 5 minutes at 3,000 rpm to remove insoluble substances, the supernatants were carefully transferred to 96-well plates with careful pipetting to avoid cross-contamination between samples. After stabilization at room temperature for 10 min to eliminate temperature-induced fluctuations in absorbance, absorbance was measured using a multifunctional microplate reader at 593 nm for Fe^{2+} and 532 nm for MDA; standard curves were constructed using gradient-diluted standard solutions provided in the kits to accurately calculate the concentration of Fe^{2+} and MDA in the samples, which respectively reflect the degree of intracellular iron accumulation and lipid peroxidation associated with ferroptosis.

Propidium Iodide (PI) staining for cell viability detection

Cells were seeded in 24-well plates paved with sterile coverslips and cultured until cell adherence. After corresponding treatments, the culture medium was discarded, and the cells were gently washed twice with pre-cooled PBS to remove residual impurities. A total of 500 μL of PI working solution (5 μg/mL, prepared with PBS) was added to each well to completely cover the cells, followed by incubation in the dark for 15 min at 37°C in an incubator. The PI solution was then discarded, and the cells were washed three times with PBS to remove unbound dye. Subsequently, the cells were fixed with 4% paraformaldehyde, and 500 μL of DAPI solution (1 μg/mL) was added for nuclear counterstaining. The cells were incubated in the dark for 10

min at 37°C in an incubator. After another three rinses with PBS, the coverslips were mounted onto glass slides with anti-fade mounting medium. Fluorescent signals were collected using a confocal microscope: red fluorescence represented PI-positive dead cells with damaged cell membranes, and blue fluorescence labeled the cell nuclei. ImageJ software was used to calculate the proportion of red fluorescent cells relative to the total number of blue-stained nuclei for the quantification of the PI-positive cell rate.

Reactive oxygen species (ROS) detection

C11 BODIPY 581/591 lipid peroxidation fluorescent probe is a lipid soluble ratiometric fluorescent probe that can enter the cardiomyocyte membrane to indicate the lipid peroxidation and antioxidant properties of the membrane system and living cells. The emission wavelength is 591 nm (non oxidized state) and 510 nm (oxidized state) in the visible region of the electromagnetic spectrum. Using confocal fluorescence microscopy, the oxidation of polyunsaturated m-butanedienyl group of dye will lead to the shift of fluorescence emission peak from 591 nm to 510 nm. The fluorescence ratio measured under 591 nm/510 nm emission light reflects the level of cellular lipid peroxidation.

Cell viability assay

Primary cardiomyocytes from neonatal mice were isolated and cultured and inoculated into 96 well plates, and then cultured in groups. Only DMEM/F12 medium was used as the blank group and cultured at 37°C in constant temperature and normoxia, while DMEM/F12 medium was used as the control group. After the cells were collected, the absorbance was detected at 593 nm according to the method of cell counting kit-8 (CCK-8) with a full-function microplate detector to determine the cell survival rate.

RNA pull-down

The extracted protein supernatants were divided into the IgG group and the

HNRNPA2B1 group. The remaining supernatant was equally divided into two tubes, designated as the IgG and HNRNPA2B1 groups. Five microliters of IgG and 5 μL of HNRNPA2B1 antibody (both IP-grade) were added to the respective groups, followed by incubation with rotation at 4°C for over 12 h. After incubation, streptavidin magnetic beads (blocked with yeast tRNA and BSA) were added to each group, and rotation was continued at 4°C for 4 h to couple proteins with the beads. Using a magnetic rack, the beads were separated from the liquid, then washed five times with high-salt RNA pull-down eluent to remove nonspecific binding, with the supernatant discarded after each wash. Low-salt RNA pull-down eluent was added to lyse the beads at 4°C for 30 min, eluting the bound proteins into the supernatant. Protein loading buffer was added, and proteins were denatured in a 95°C metal bath for 7 min. The interaction between the target RNA and protein was detected via Coomassie brilliant blue staining. A portion of cell suspension was ground in an precooled glass grinder for 30 min.

RNA immunoprecipitation (RIP) assay

Lysates were centrifuged (12,000 rpm, 20 min, 4°C), and RNA-containing supernatant was collected. A 50 μL aliquot was stored as input at -20°C; the rest was split into IgG and HNRNPA2B1 groups. Corresponding antibodies (5 μL each) were added, incubated with rotation at 4°C for 12 h. Prepared protein A/G agarose microspheres (60 μL) were added to each group, rotated at 4°C for 4 h. After centrifugation, microspheres were washed five times with low-salt RIP eluent. Trizol (1000 μL) was added to all groups for RNA extraction to detect protein-RNA binding.

Mitochondrial probe mitotracker staining

Mitochondrial staining was performed using MitoTracker Red CMXRos, an oxidized red fluorescent dye specifically designed for labeling mitochondria in living cells. This dye binds stably to mitochondria even after fixation and permeabilization, making it suitable for subsequent co-staining experiments; its red fluorescence

(excitation wavelength: 579 nm; emission wavelength: 599 nm) allows visualization of mitochondrial distribution and structural changes.

Prior to staining, MitoTracker Red CMXRos powder was equilibrated to room temperature and dissolved in cell culture-grade anhydrous DMSO to prepare a 1 mM stock solution. The stock solution was aliquoted, stored at -20°C in the dark to avoid repeated freeze-thaw cycles, and then diluted to a 500 nM working solution (freshly prepared with pre-warmed complete medium, optimized to minimize artifacts and toxicity) for use. For primary cardiomyocytes cultured on coverslips, the original medium was aspirated, and the pre-warmed working solution was added to fully cover the cells. The cells were incubated at 37°C under 5% CO_2 for 40 minutes, followed by three 5-minute washes with pre-warmed PBS to remove unbound dye. The cells were then fixed with 4% paraformaldehyde at room temperature for 15 minutes, followed by three additional 5-minute PBS washes to remove residual fixative. Nuclear counterstaining was performed using a 1 μg/mL DAPI solution (incubated at room temperature in the dark for 10 minutes), and excess DAPI was removed by three final PBS washes. Coverslips were mounted using anti-fade mounting medium, and fluorescent signals were detected using a Leica inverted two-photon laser confocal scanning microscope. The red fluorescent signal corresponds to the location and morphology of mitochondria, while the blue signal indicates cell nuclei.

To assess the degree of mitochondrial fission, the morphological analysis tool Analyze Particles in ImageJ was used for quantitative detection: the longest and shortest axis lengths of individual mitochondria were measured, and the length-width ratio was calculated. Fragmented mitochondria exhibit a significantly reduced length-width ratio (≤2 is defined as fragmentation), and the percentage of mitochondria with length-width ratio ≤2 relative to the total number of mitochondria was quantified for each experimental group.

One-step TUNEL cell apoptosis detection

Apoptosis was assessed using the One-step TUNEL Cell Apoptosis Detection Kit

(Red, Dye 555, Cat. No. CX108, CellorLab, Shanghai) following the manufacturer's protocol.The TUNEL reaction mixture was freshly prepared on ice by combining 45 μL Advanced 555 Labeling Mixture with 5 μL Recombinant TdT Enzyme per sample. A negative control was included by omitting the TdT enzyme. Each sample was incubated with 50 μL of the reaction mixture in a humidified chamber at 37°C for 60 min in the dark. After three washes with PBS, samples were stained with cTNT primary antibody and a green-fluorescent secondary antibody, then counterstained with DAPI for nuclei, and mounted with anti-fade mounting medium. Red TUNEL signals (Ex/Em: 554/567 nm), green cTNT signals, and blue DAPI nuclear staining were visualized and analyzed using a fluorescence microscope.

Mitochondrial isolation and protein extraction

Cultured cells or fresh animal tissues were processed using the Proteintech Mitochondrial Isolation and Protein Extraction Kit (Cat. No. PK10016). Cells were harvested, washed with PBS, and resuspended in ice-cold Reagent A at 1 mL per 20 million cells. Tissues were minced and homogenized in Reagent A at 1 mL per 100 mg tissue. Samples were homogenized on ice, with efficiency monitored by 0.4% trypan blue staining. The homogenate was overlaid onto Reagent B and centrifuged at 2600 rpm for 10 min at 4°C. The upper layer was collected and centrifuged at 10600 rpm for 10 min to obtain crude mitochondria. For purification, crude mitochondria were layered onto a 17:3 mixture of Separation Solution C and D, centrifuged at 15700 rpm for 10 min, and washed at 13400 rpm for 5 min at 4°C. Purified mitochondria were verified with Janus Green B staining and used for functional assays or lysed for protein analysis.

Construction of adenovirus

HNRNPA2B1 overexpression adenovirus and Fis1 overexpression adenovirus were constructed by the Genechem Company (Shanghai, China). The principle is as follows: Linearized vectors are obtained by digestion with restriction endonucleases.

The target gene fragment was prepared by PCR amplification. The amplification primers used need to have homologous recombination sequences added at their 5 'ends during design (marked in green and blue in the figure). When using this primer to amplify the target gene fragment, the sequences at the 5' and 3 'ends of the amplification product are completely consistent with the sequences at the two ends of the linearized cloning vector, respectively. Prepare the reaction system for the gene amplification products, carry out the recombination reaction, and achieve the in vitro cyclization of the linearized vector and the target gene fragment. The recombinant products were directly transformed. The single clones on the plate were picked for PCR identification, and the positive clones were sequenced and the results were analyzed. The correct cloned bacterial liquid was expanded for culture and extraction to obtain high-purity plasmids for downstream virus packaging.

Assessment of mRNA stability treated with actinomycin D

To evaluate the stability of Fis1 mRNA in cardiomyocytes, primary cardiomyocytes in the logarithmic growth phase were seeded into 6-well plates. When cell confluency reached 70–80%, cells were divided into two groups: the NC group and the FERPIR overexpression group (FERPIR overexpression was achieved via agomir transfection, following the protocol described previously). Both groups were treated with actinomycin D (final concentration: 0.1 μg/mL) to block de novo RNA synthesis, and cell samples were collected at 0 h, 2 h, 4 h, and 6 h post-administration. After washing twice with pre-cooled PBS at each time point, total RNA was extracted using TRIzol reagent, and RNA concentration and purity (A260/A280 ratio: 1.8–2.1) were determined via Nanodrop. Subsequently, 1 μg of RNA was reverse-transcribed into cDNA using a reverse transcription kit, and RT-qPCR was performed with cDNA as the template to detect the expression level of Fis1 mRNA. The relative residual level of Fis1 mRNA at each time point was calculated using the 0 h level as the baseline, and a degradation kinetic curve was plotted. The half-life of Fis1 mRNA was analyzed to assess differences in its stability between groups.
