# Supplementary figures and images for: FERPIR promotes cardiomyocyte survival and attenuates cardiac remodeling after myocardial infarction
Source: Cell Death Dis. 2026 May 21;17(1):639. doi: 10.1038/s41419-026-08817-8 (PMC13365201; doi:10.1038/s41419-026-08817-8)

**A**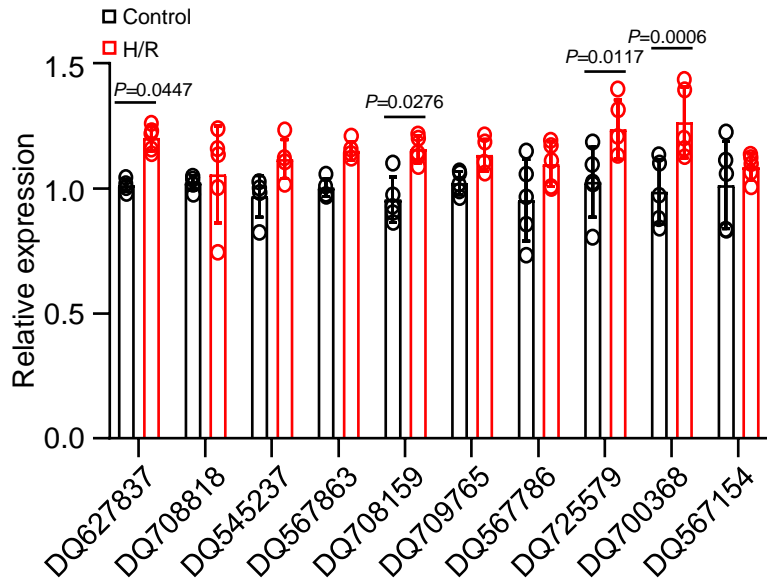**B**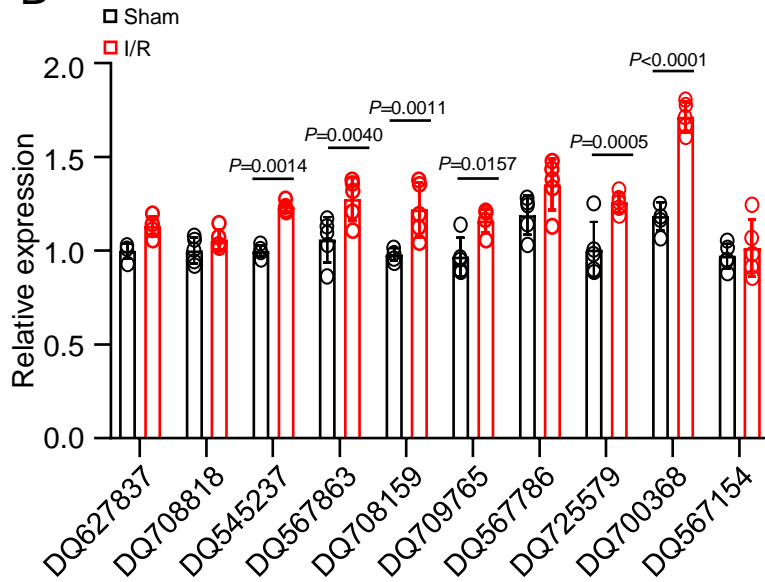**C**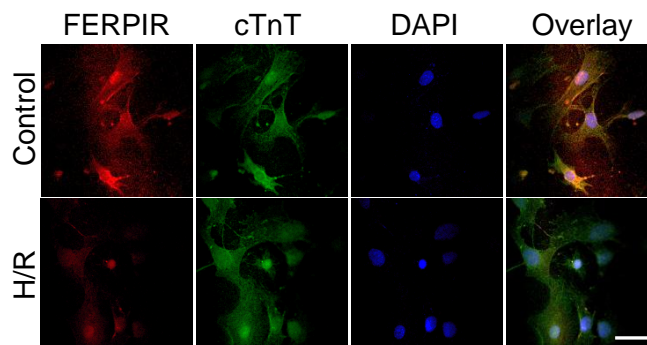

A

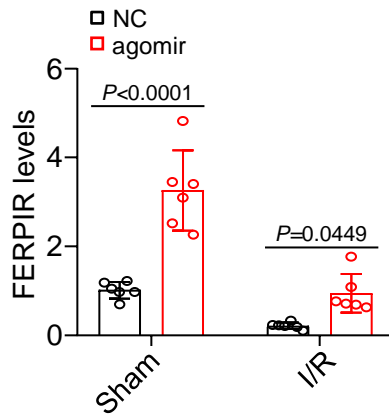

B

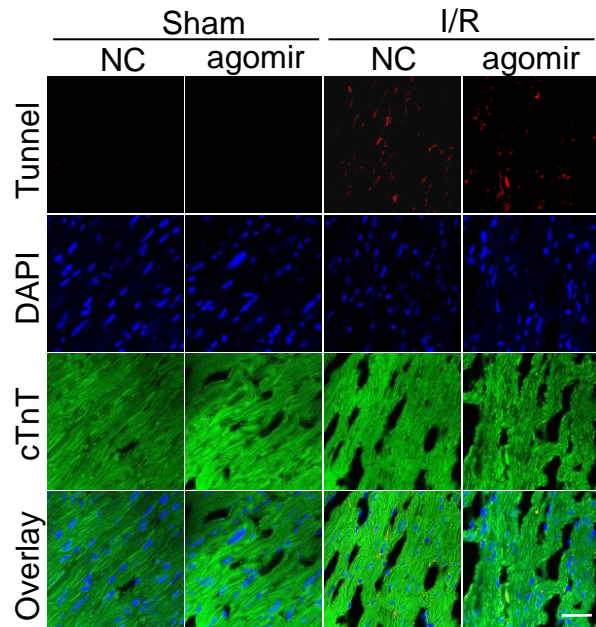

C

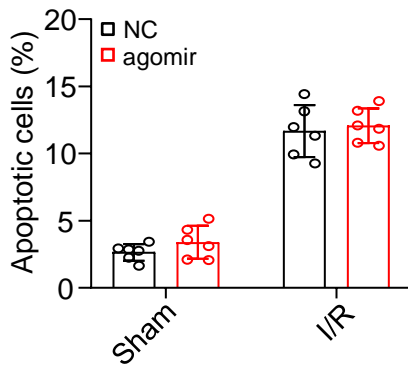

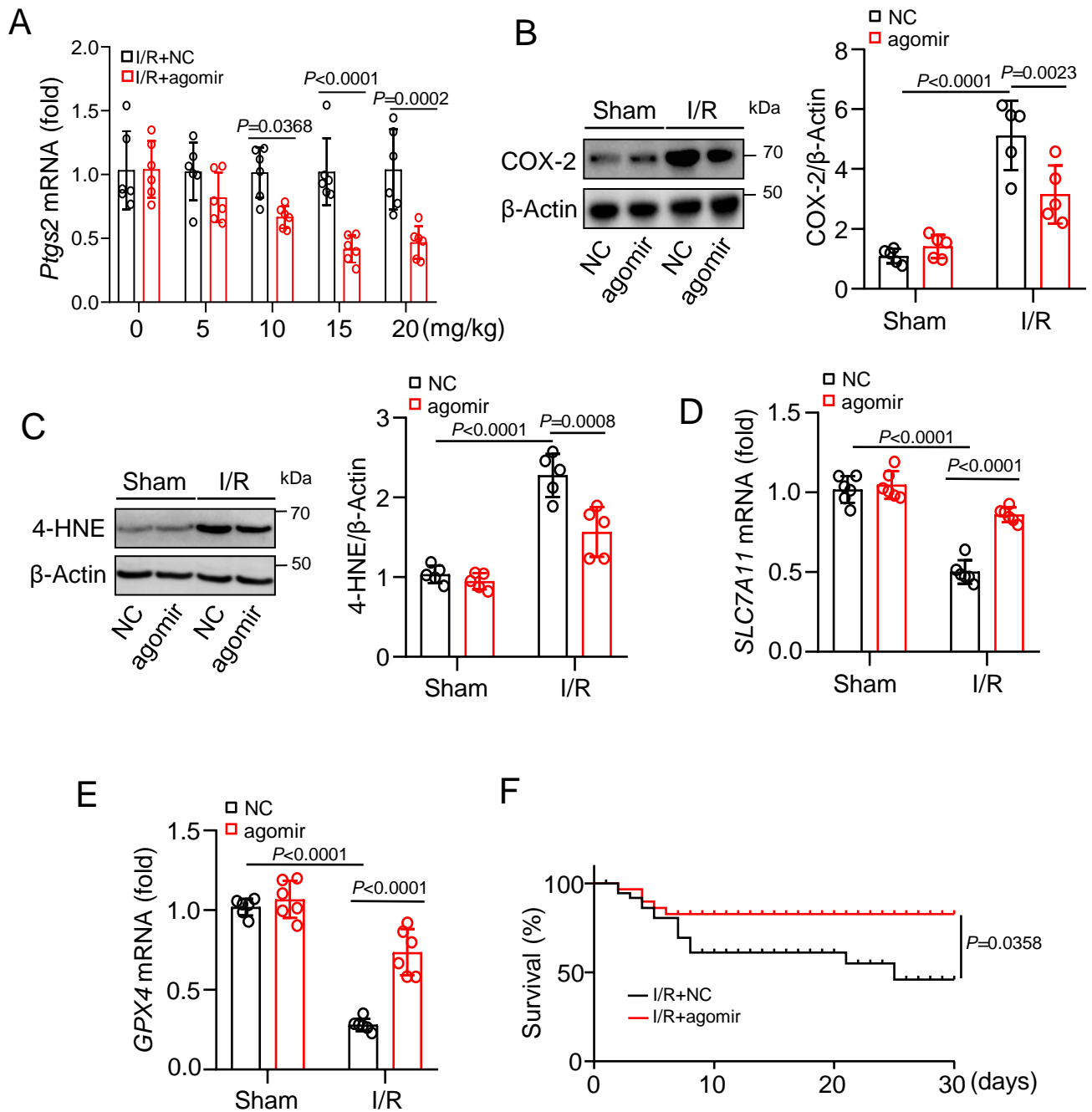

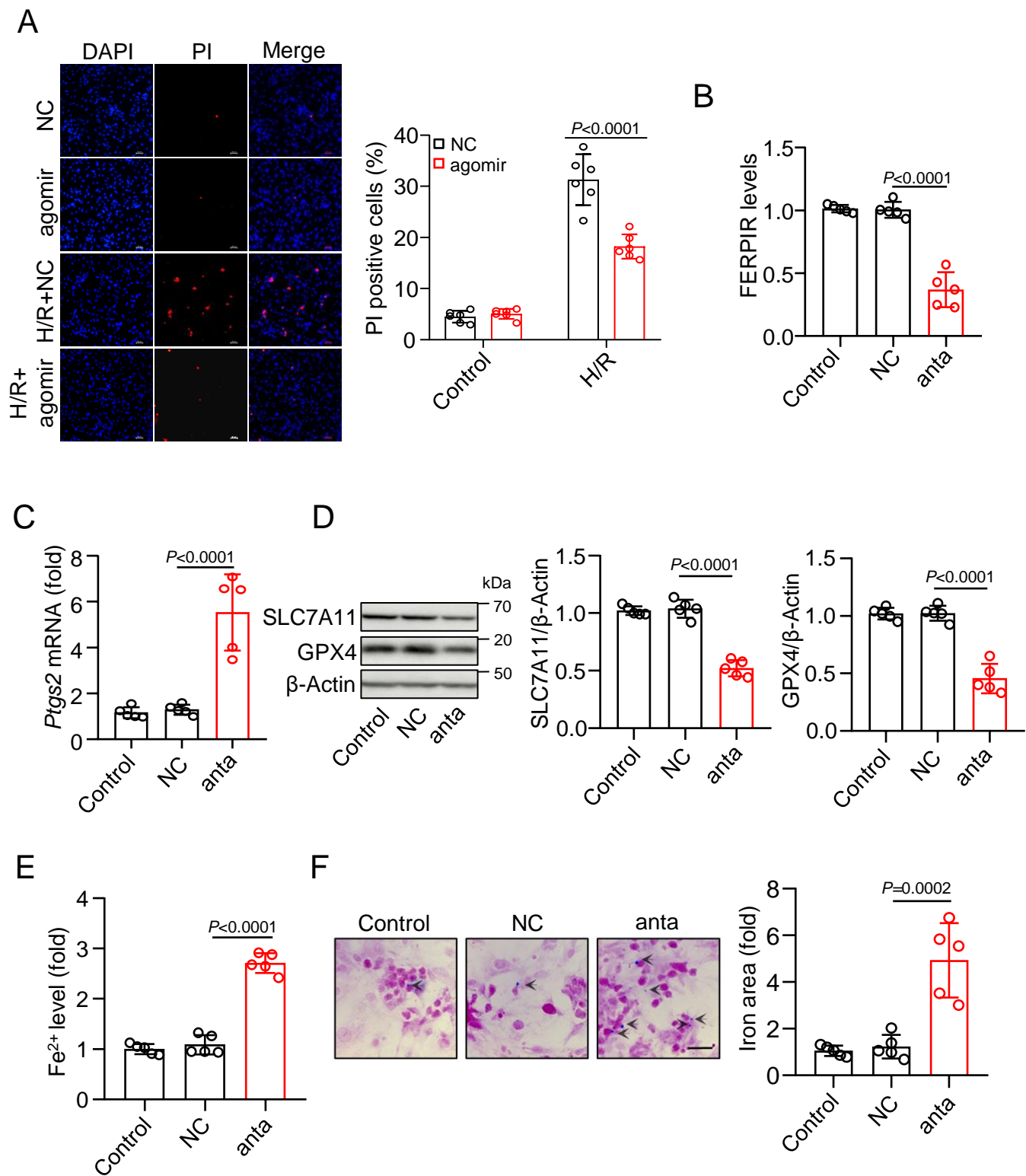

A

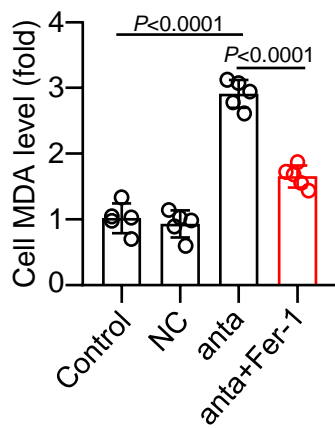

B

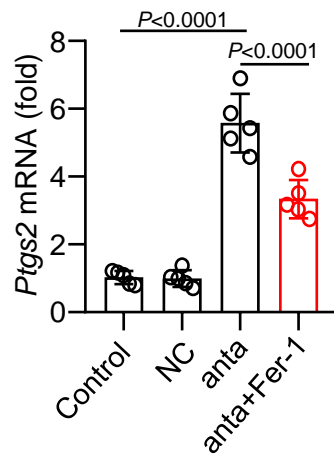

C

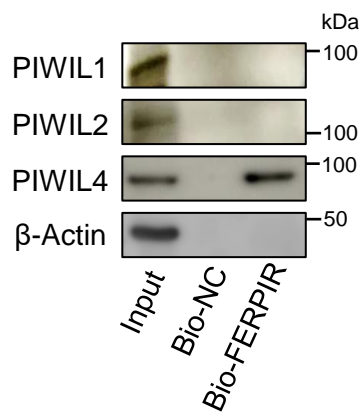

D

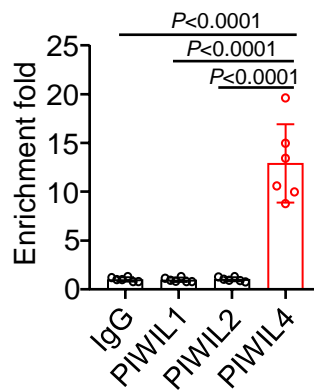

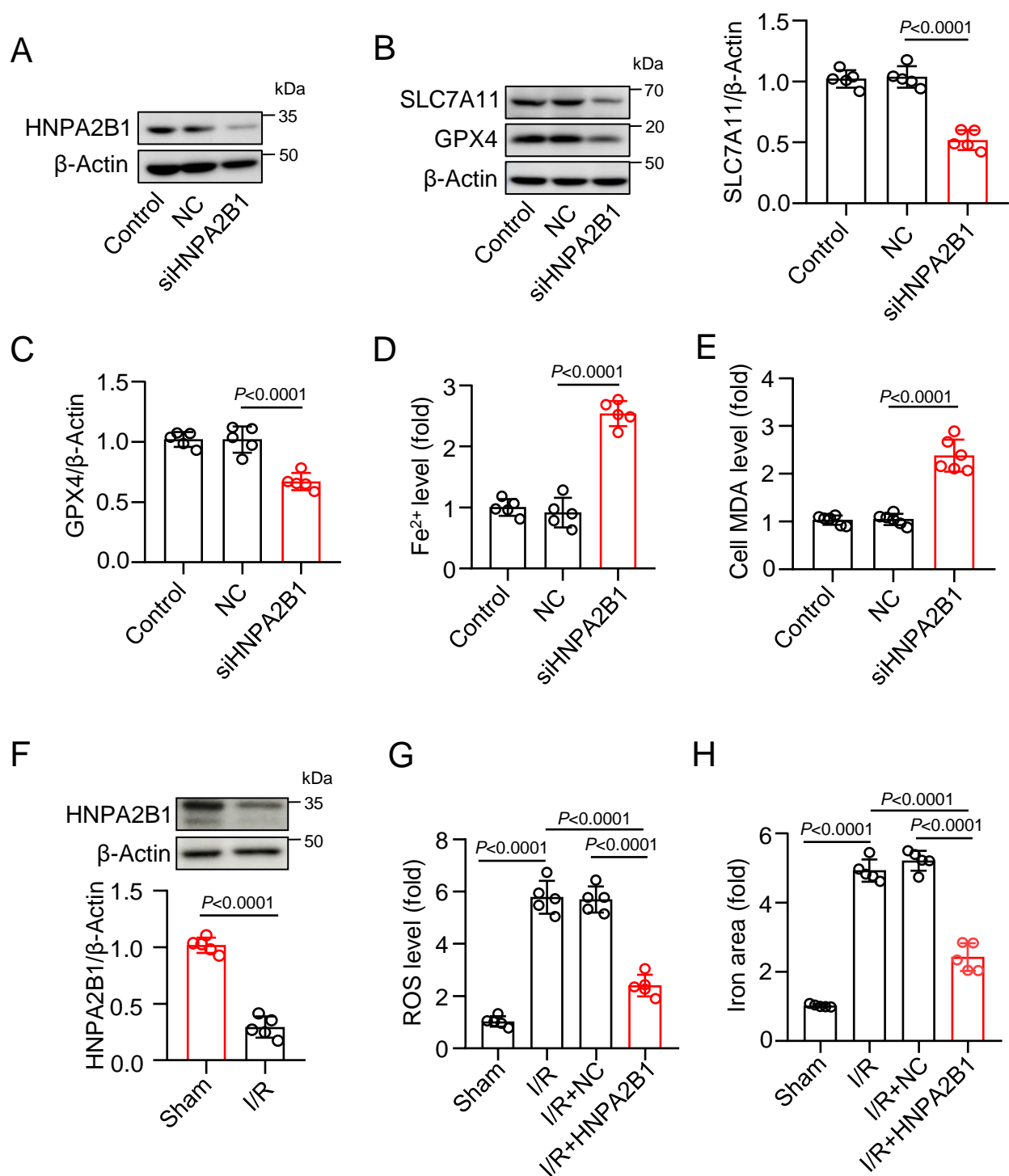

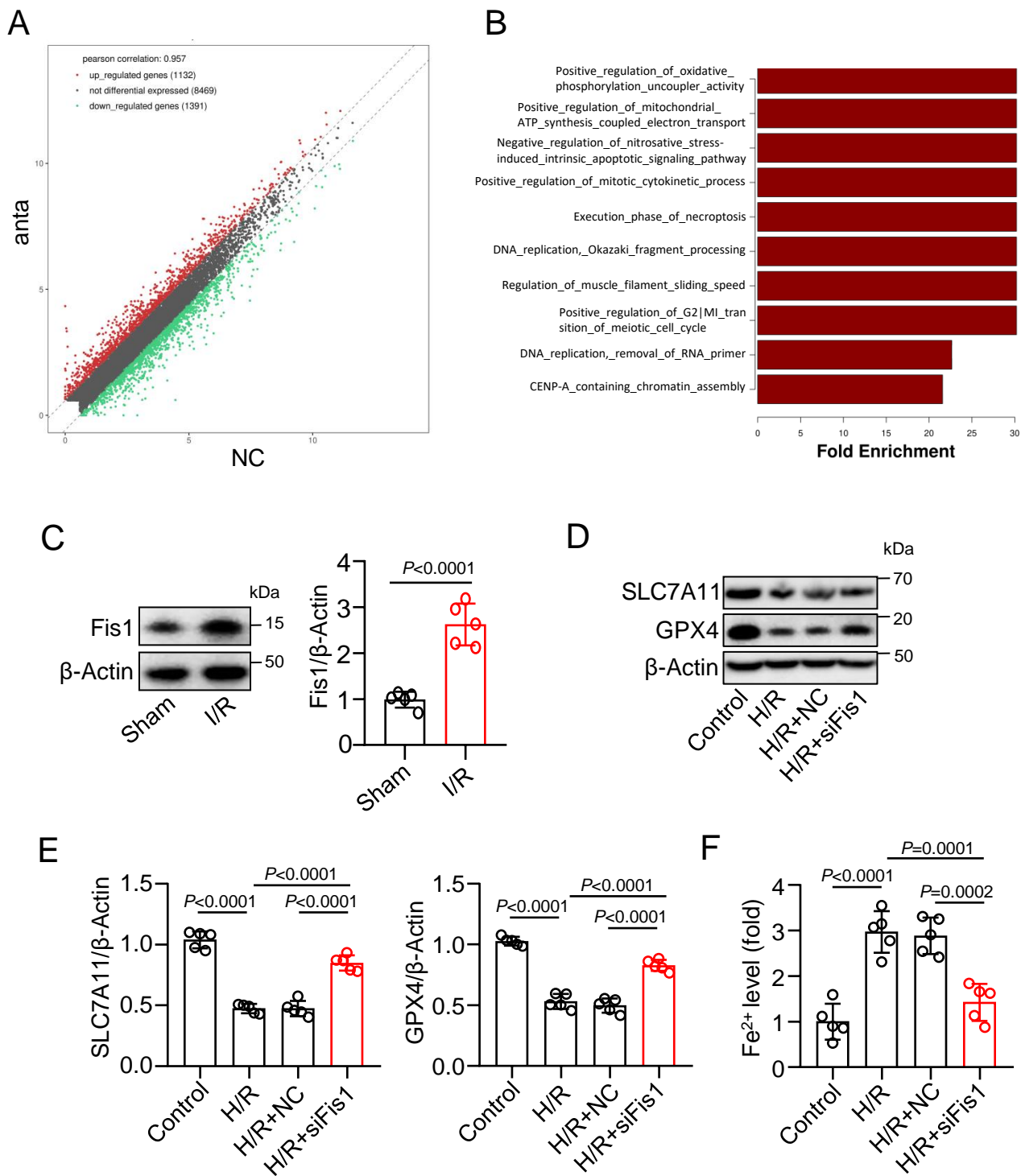

A

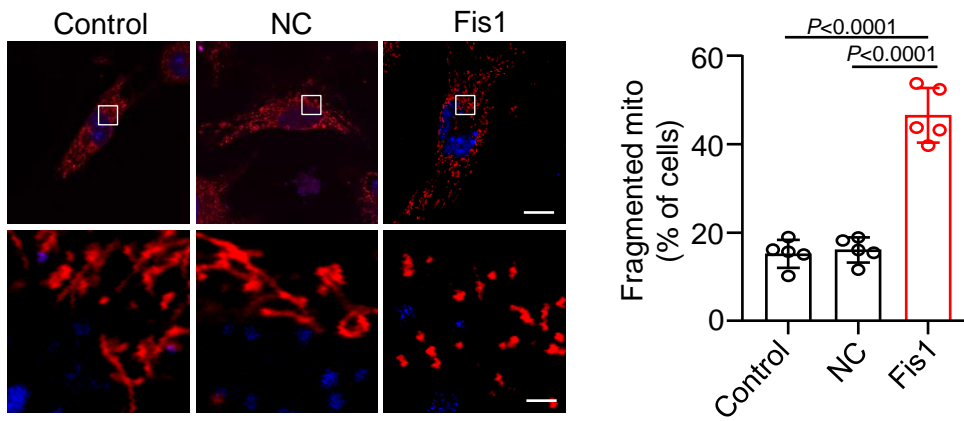

B

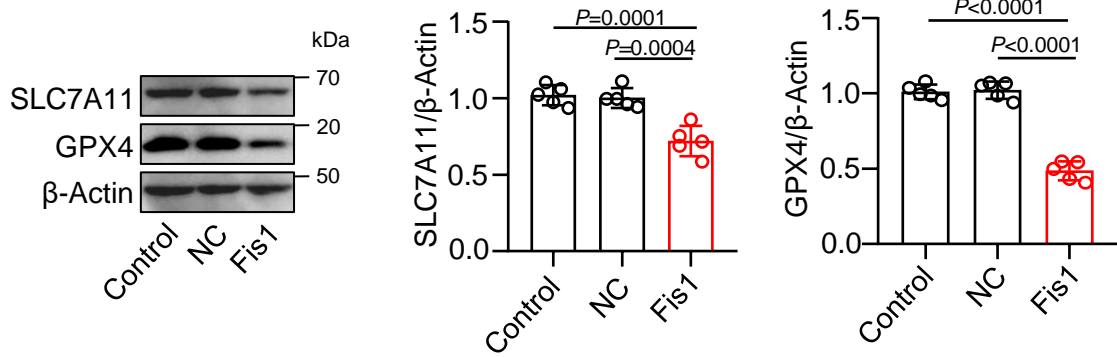

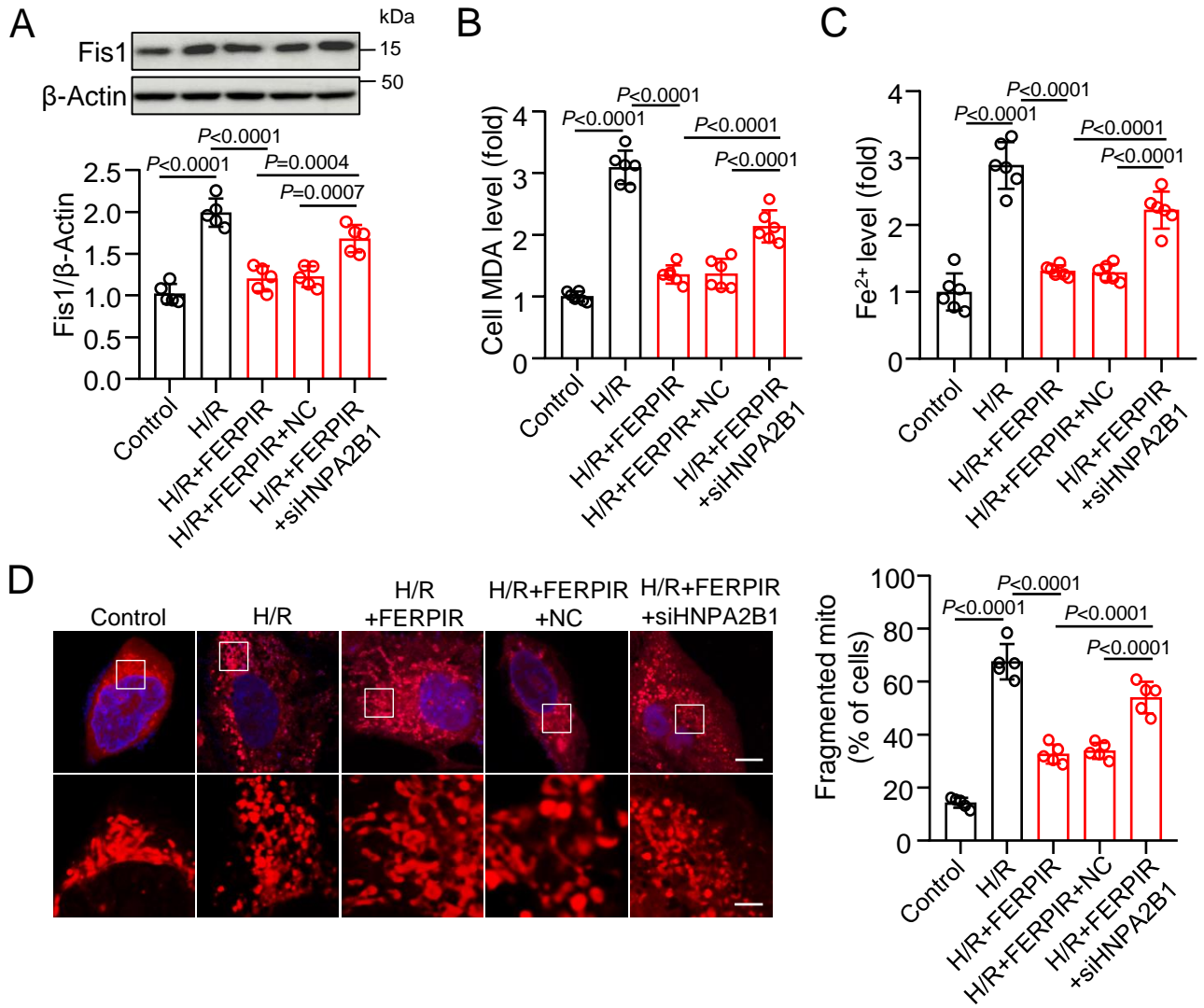

Supplement: Supplementary file 2 — Supplementary figure [file 41419_2026_8817_MOESM2_ESM.pdf]
