# Supplementary material for: FERPIR promotes cardiomyocyte survival and attenuates cardiac remodeling after myocardial infarction
Source: Cell Death Dis. 2026 May 21;17(1):639. doi: 10.1038/s41419-026-08817-8 (PMC13365201; doi:10.1038/s41419-026-08817-8)
Supplement: Supplementary file 3 — Supplementary material legends [file 41419_2026_8817_MOESM3_ESM.pdf]

SUPPLEMENTARY MATERIAL LEGENDS

Supplementary Figure 1. Screening of piRNAs. (A) Relative expression levels of 10 upregulated candidate piRNAs were analysed by RT-qPCR in the control and H/R treated cardiomyocytes (n=5 independent experiments). **(B)** Relative expression levels of 10 upregulated candidate piRNAs were analysed by RT-qPCR in the hearts of mice from the sham and I/R groups (n=5 mice per group). **(C)** Fluorescence in situ hybridization and immunofluorescence co-localization images showing the expression and localization of FERPIR in cardiomyocytes. FERPIR was labeled with a probe (red), cardiomyocytes were labeled with cardiac troponin T (cTnT, green), and cell nuclei were labeled with DAPI (blue). Scale bar, 45 μm. Data are presented as mean ± SD, and statistical differences were analyzed using two-way ANOVA followed by Sidak's multiple comparison test (Supplementary Figures 1A and B). A *P*-value < 0.05 was considered statistically significant.

Supplementary Figure 2. Overexpression of FERPIR does not affect cell apoptosis. (A) Mice were injected intravenously with FERPIR-agomir or a negative control (FERPIR-NC). Three days post-injection, animals underwent either sham surgery or left anterior descending coronary artery ligation for 30 minutes, followed by 24 hours of reperfusion. Cardiomyocytes were isolated from the heart tissues of mice in the sham and I/R groups. Relative expression levels of FERPIR was detected by RT-qPCR (n=6 mice per group). **(B)** Immunofluorescence staining images showing the effect of agomir on apoptosis. Apoptotic cells were stained with TUNEL (red fluorescence), cardiomyocytes were labeled with cTnT (green fluorescence), cell nuclei were labeled with DAPI (blue fluorescence). Scale bar, 40 μm. **(C)** Quantifying the proportion of apoptotic cells (n=6 mice per group). Data are presented as mean ± SD, and statistical differences were analyzed using two-way ANOVA followed by Sidak's multiple comparison test (Supplementary Figures 2A and C). A *P*-value < 0.05 was considered statistically significant.

Supplementary Figure 3. Overexpression of FERPIR alleviates I/R-induced ferroptosis. (A) Dose-gradient experiment of FERPIR agomir (agomir). After injection of agomir at doses of 0, 5, 10, 15, and 20 mg/kg, the relative expression level of *Ptgs2* mRNA was detected by RT-qPCR (n=6 mice per group). **(B)** Western blot analysis and quantification of COX-2 protein expression, with β-Actin used as an internal control (n=5 mice per group). **(C)** Western blot analysis and quantification of 4-HNE protein (a marker of oxidative stress) expression, with β-Actin used as an internal control (n=5 mice per group). **(D)** Relative expression level of *SLC7A11* mRNA, was detected by RT-qPCR (n=6 mice per group). **(E)** Relative expression level of *GPX4* mRNA was detected by RT-qPCR (n=5 mice per group). **(F)** Kaplan-Meier survival curve showing the 30-day survival rate of the I/R+agomir group (n=37 mice per group) and the I/R+NC group (n=29 mice per group). Data are presented as mean ± SD; statistical differences for quantitative data were analyzed using two-way ANOVA followed by Sidak's multiple comparison test (Supplementary Figures 3A-E), and differences in survival curves were analyzed using the Log-rank test (Supplementary Figure 3F). A P-value < 0.05 was considered statistically significant.

Supplementary Figure 4. Inhibition of FERPIR enhances ferroptosis. (A) Cardiomyocytes were transfected with FERPIR agomir (agomir), and fluorescence staining images showed the effect of agomir on cell death. Dead cells were stained with Propidium Iodide (PI, red fluorescence), cell nuclei were labeled with DAPI (blue fluorescence). Scale bar, 25 μm. Bar graph quantifying the proportion of PI-positive cells (n=6 independent experiments). **(B)** Cardiomyocytes were transfected with FERPIR antagomirs (anta), relative expression levels of FERPIR was detected by RT-qPCR (n=5 independent experiments). **(C)** Relative expression level of *Ptgs2* mRNA was detected by RT-qPCR (n=5 independent experiments). **(D)** Western blot analysis and quantification of SLC7A11 and GPX4 protein expression

were performed, with β-Actin used as an internal control (n=5 independent experiments). **(E)** Quantifying the proportion of Fe^{2+} levels in cardiomyocytes (n=5 independent experiments). **(F)** Prussian blue staining reflecting the degree of iron deposition in cardiomyocytes, with blue spots indicating iron deposition areas (n=5 independent experiments). Scale bar, 25 μm. Data are presented as mean ± SD, and statistical differences were analyzed using one-way ANOVA followed by Tukey's multiple comparison test (Supplementary Figures 4B-F), statistical differences for quantitative data were analyzed using two-way ANOVA followed by Sidak's multiple comparison test (Supplementary Figure 4A). A *P*-value < 0.05 was considered statistically significant.

Supplementary Figure 5. Fer-1 reverses cell damage induced by FERPIR antagomirs. (A) Cardiomyocytes were pretreated with 1 μM Fer-1 for 12 hour, followed by transfection with FERPIR antagomirs (anta). Quantifying the proportion of MDA levels (n=5 independent experiments). **(B)** Relative expression level of *Ptgs2* mRNA was detected by RT-qPCR (n=5 independent experiments). **(C)** Pull-down assay followed by Western blot to detect the binding of PIWIL1, PIWIL2, or PIWIL4 with FERPIR. β-Actin was used as an internal control. **(D)** RIP assay using PIWIL1, PIWIL2 or PIWIL4 antibodies for detection and quantification of FERPIR enrichment fold (n=6 independent experiments). Data are presented as mean ± SD, and statistical differences were analyzed using one-way ANOVA followed by Tukey's multiple comparison test (Supplementary Figures 5A, B and D). A *P*-value < 0.05 was considered statistically significant.

S**upplementary Figure 6. HNPA2B1 is involved in the regulation of ferroptosis. (A)** Cardiomyocytes were transfected with siHNPA2B1. Western blot analysis and quantification of HNPA2B1 protein expression were performed, with β-Actin used as an internal control. **(B** and **C)** Western blot analysis and quantification of SLC7A11 and GPX4 protein expression were performed, with β-Actin used as an internal

control (n=5 independent experiments). **(D)** Quantifying the proportion of Fe^{2+} levels in cardiomyocytes (n=5 independent experiments). **(E)** Quantifying the proportion of MDA levels (n=5 independent experiments). **(F)** Western blot analysis and quantification of HNPA2B1 protein expression in cardiac tissues of mice from the sham and I/R groups, with β-Actin used as an internal control (n=5 mice per group). **(G)** Mice were injected with adenovirus to overexpress HNPA2B1 and subsequently subjected to I/R injury. Lipid peroxidation fluorescent probe was used to detect and quantify ROS levels (n=5 mice per group). **(H)** Mice were treated as described in (G). Prussian blue staining reflecting the degree of iron deposition in cardiac tissue (n=5 mice per group). Data are presented as mean ± SD, and statistical differences were analyzed using one-way ANOVA followed by Tukey's multiple comparison test (Supplementary Figures 6B-E, G and H) or unpaired Student's t-test (Supplementary Figure 6F). A *P*-value < 0.05 was considered statistically significant.

Supplementary Figure 7. Knockdown of Fis1 alleviates ferroptosis. (A) RNA-seq scatter plot. Cardiomyocytes were transfected with FERPIR antagomir (anta) and its negative control (NC) for 48h. **(B)** Gene Ontology (GO) biological process enrichment analysis of differentially expressed genes. **(C)** Western blot analysis and quantification of Fis1 protein expression in cardiac tissues of mice from the sham and I/R groups, with β-Actin used as an internal control (n=5 mice per group)**. (D** and **E)** Cardiomyocytes were transfected with siFis1, and then treated with H/R. Western blot analysis and quantification of SLC7A11 and GPX4 protein expression were performed, with β-Actin used as an internal control (n=5 independent experiments). **(F)** Quantifying the proportion of Fe^{2+} levels in cardiomyocytes (n=5 independent experiments). Data are presented as mean ± SD, and statistical differences were analyzed using one-way ANOVA followed by Tukey's multiple comparison test (Supplementary Figures 7E and F) or unpaired Student's t-test (Supplementary Figure 7C). A *P*-value < 0.05 was considered statistically significant.

Supplementary Figure 8. Overexpression of Fis1 induces ferroptosis. (A) Cardiomyocytes were transfected with Fis1 or NC for 48h. MitoTracker Red CMXRos (red fluorescence, labeling mitochondria in living cells) was used for staining, and DAPI (blue fluorescence, labeling cell nuclei) was used for nuclear counterstaining; the upper panel shows low-magnification fields (white boxes indicate high-magnification regions), and the lower panel shows high-magnification fields. Scale bars=20 μm (low magnification) and 4 μm (high magnification). Bar graph quantifying the fluorescence images showed the proportion of fragmented mitochondria (n=5 independent experiments). **(B)** Western blot analysis and quantification of SLC7A11 and GPX4 protein expression were performed, with β-Actin used as an internal control (n=5 independent experiments). Data are presented as mean ± SD, and statistical differences were analyzed using one-way ANOVA followed by Tukey's multiple comparison test (Supplementary Figures 8A and B). A P-value < 0.05 was considered statistically significant.

Supplementary Figure 9. FERPIR modulates mitochondrial fission and cardiomyocyte ferroptosis by targeting HNPA2B1/Fis1. AC16 cells were transfected with FERPIR agomir (agomir), siHNPA2B1 or NC, and then treated with H/R. **(A)** Western blot analysis and quantification of Fis1 protein in AC16 cardiomyocytes, with β-Actin used as an internal control (n=5 independent experiments). **(B)** Quantifying the proportion of MDA levels in AC16 cells (n=6 independent experiments). **(C)** Quantifying the proportion of Fe^{2+} levels in AC16 cells (n=6 independent experiments). **(D)** MitoTracker Red CMXRos (red fluorescence, labeling mitochondria in living cells) was used for staining AC16 cardiomyocytes, and DAPI (blue fluorescence, labeling cell nuclei) was used for nuclear counterstaining; the upper panel shows low-magnification fields (white boxes indicate high-magnification regions), and the lower panel shows high-magnification fields. Scale bars=4 μm (low magnification) and 1 μm (high magnification). Bar graph quantifying the fluorescence images showed the proportion of fragmented

mitochondria (n=5 independent experiments). Data are presented as mean ± SD, and statistical differences were analyzed using one-way ANOVA followed by Tukey's multiple comparison test (Supplementary Figures 9A-D). A *P*-value < 0.05 was considered statistically significant.
