# Supplementary material for: FERPIR promotes cardiomyocyte survival and attenuates cardiac remodeling after myocardial infarction
Source: Cell Death Dis. 2026 May 21;17(1):639. doi: 10.1038/s41419-026-08817-8 (PMC13365201; doi:10.1038/s41419-026-08817-8)

Full unedited gel for Figure 2

Figure 2F

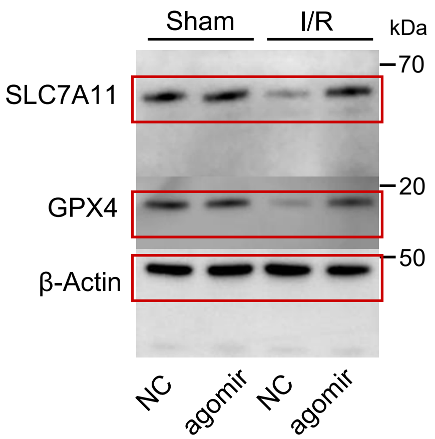

Full unedited gel for Figure 4

Figure 4E

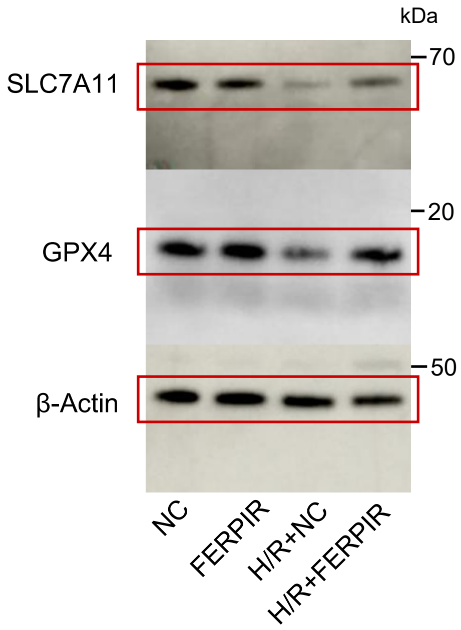

Full unedited gel for Figure 5

Figure 5B

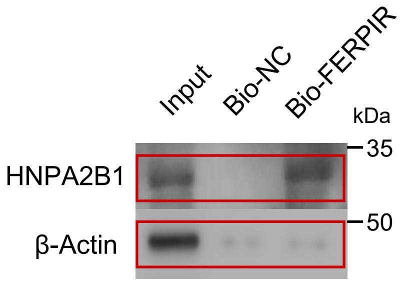

Figure 5D

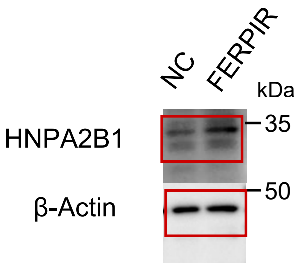

Figure 5E

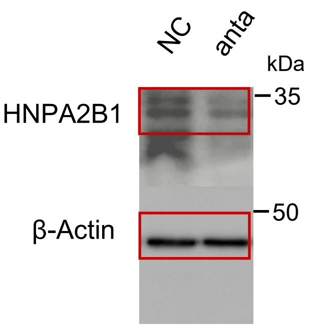

Figure 5F

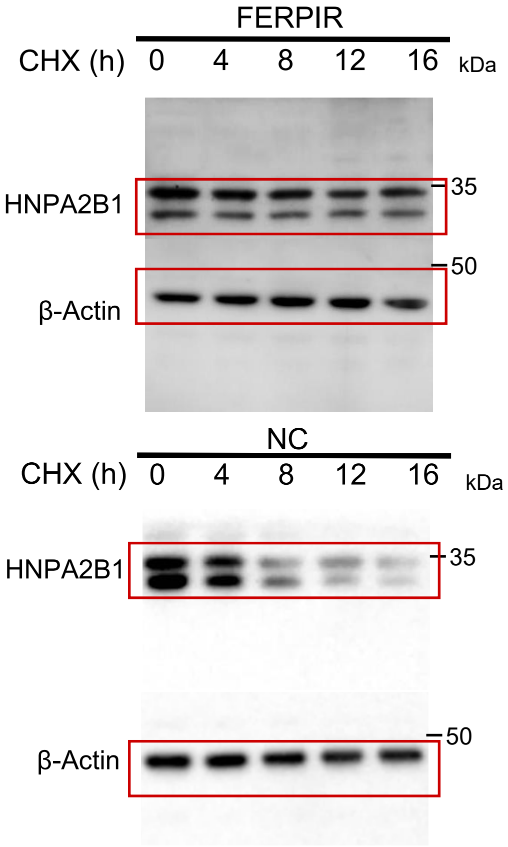

Full unedited gel for Figure 6

Figure 6A

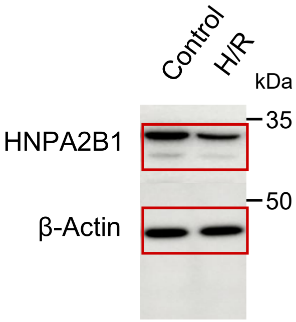

Figure 6B

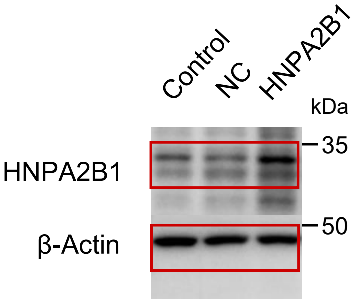

Figure 6C

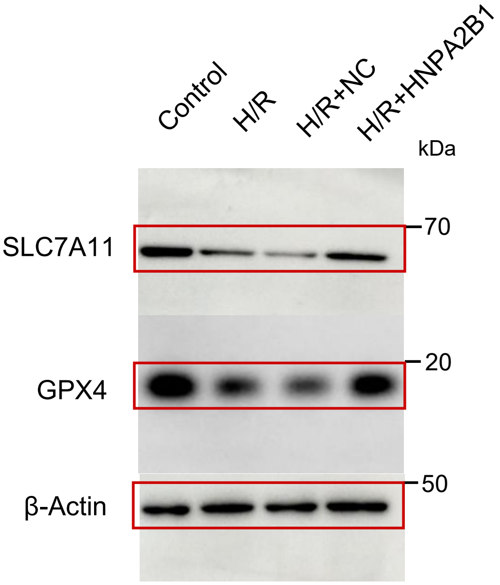

Full unedited gel for Figure 7

Figure 7B

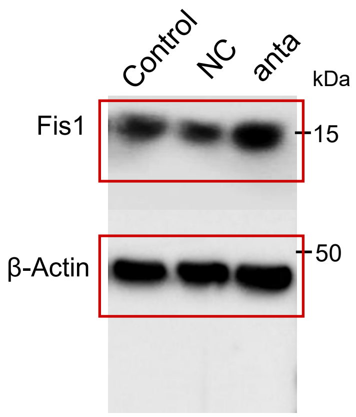

Figure 7D

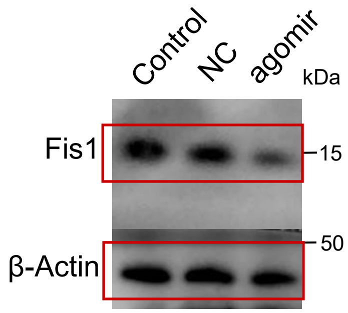

Figure 7H

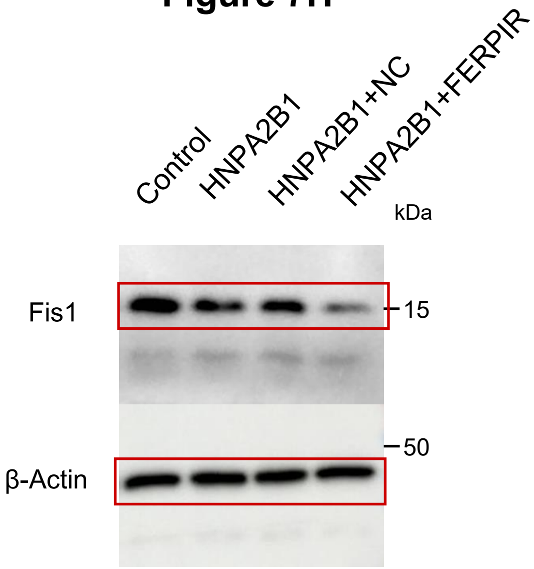

Full unedited gel for Figure 8

Figure 8C

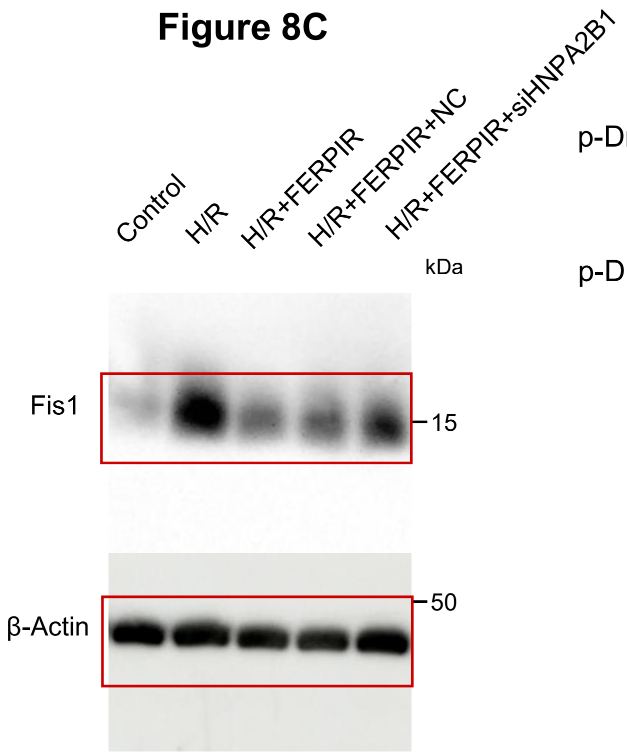

Figure 8G

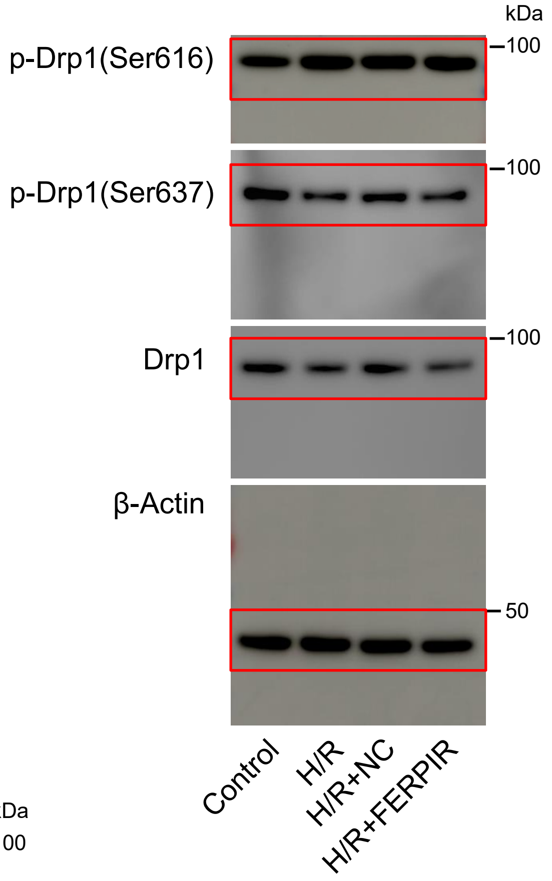

Figure 8I

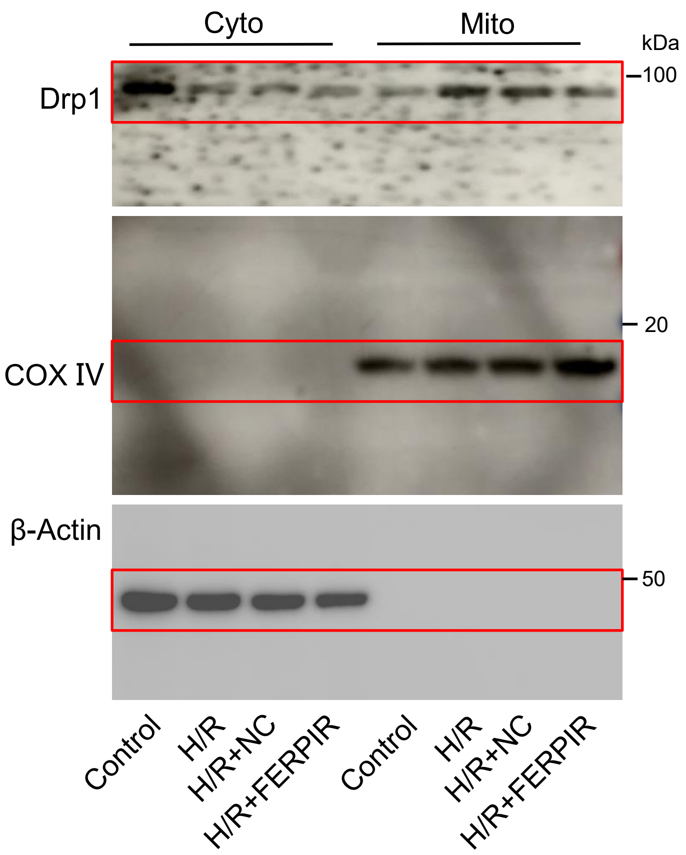

Full unedited gel for Supplementary Figure 3

Supp Figure 3B

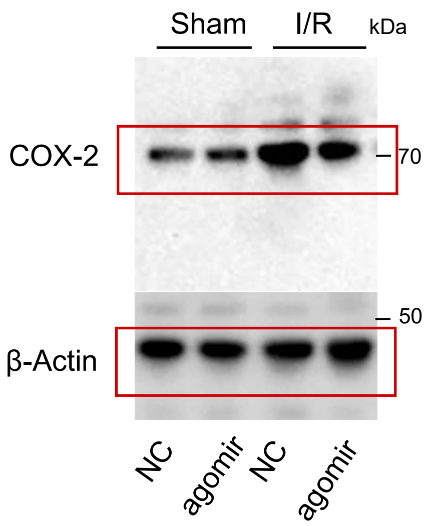

Supp Figure 3C

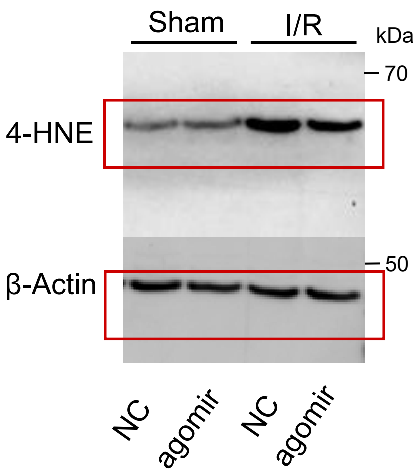

Full unedited gel for Supplementary Figure 4

Supp Figure 4D

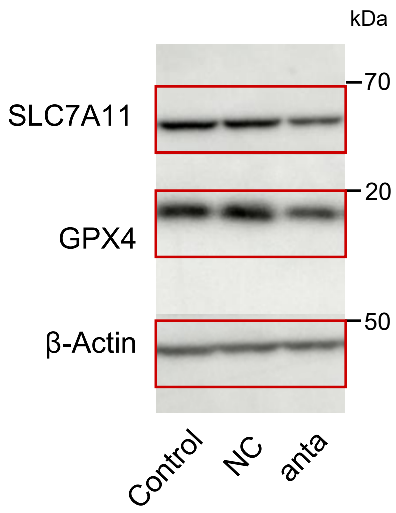

Full unedited gel for Supplementary Figure 5

Supp Figure 5C

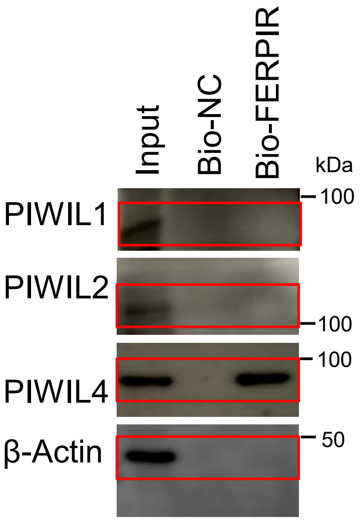

Full unedited gel for Supplementary Figure 6

Supp Figure 6A

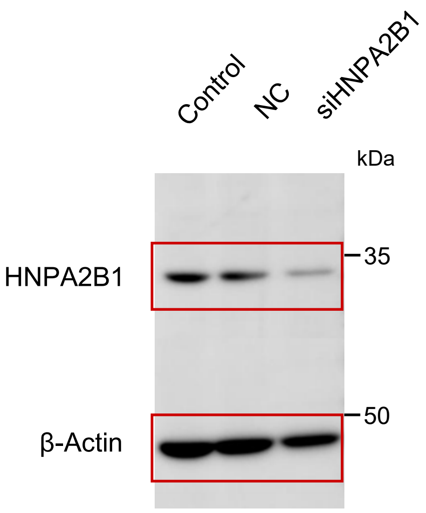

Supp Figure 6B

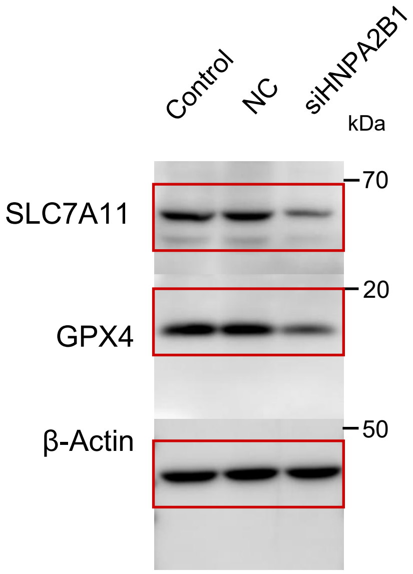

Supp Figure 6F

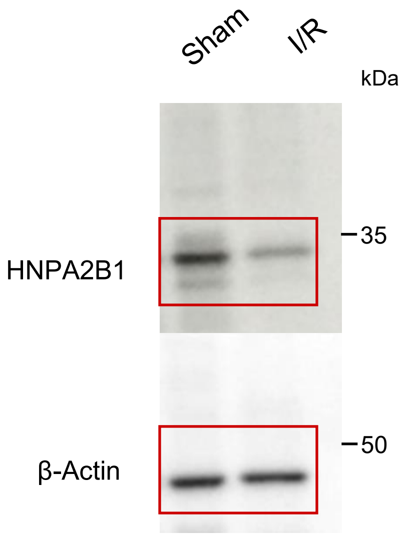

Full unedited gel for Supplementary Figure 7

Supp Figure 7C

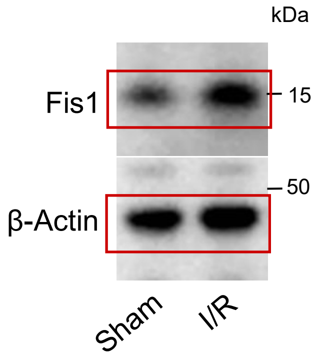

Supp Figure 7D

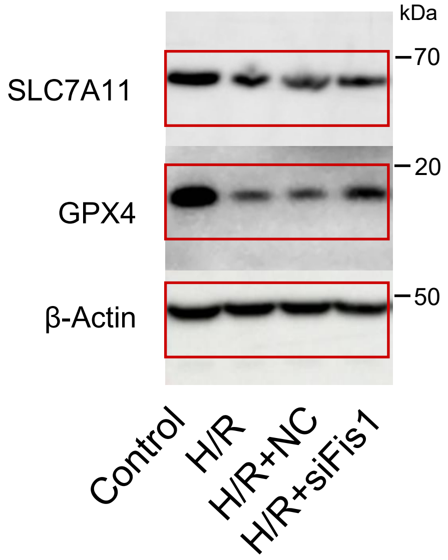

Full unedited gel for Supplementary Figure 8

Supp Figure 8B

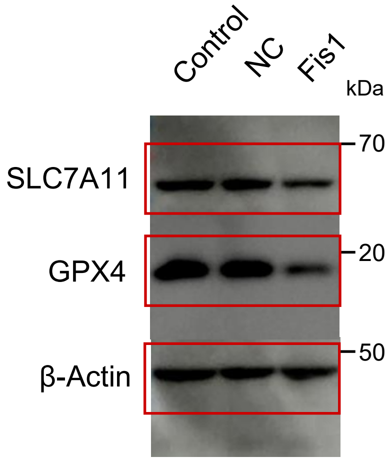

Full unedited gel for Supplementary Figure 9

Supp Figure 9A

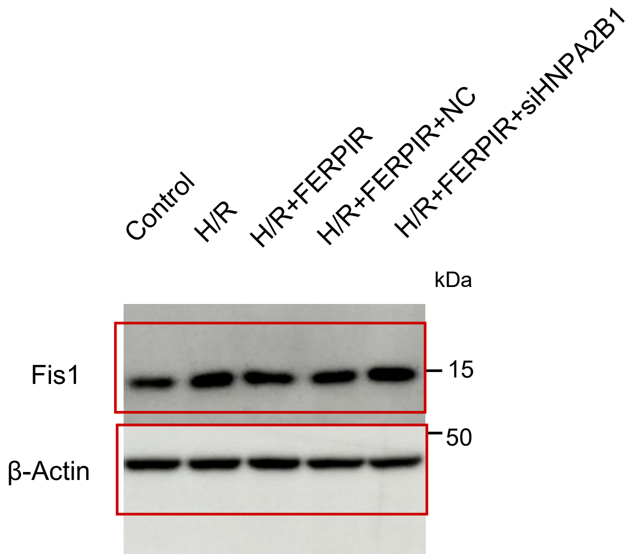

Supplement: Supplementary file 4 — Full uncropped gel [file 41419_2026_8817_MOESM4_ESM.pdf]
